# Targeting FGFR4 abrogates HNF1A-driven metastasis in pancreatic ductal adenocarcinoma

**DOI:** 10.1186/s12943-025-02408-5

**Published:** 2025-07-29

**Authors:** Katherine J. Crawford, Kennedy S. Humphrey, Eduardo Cortes, Jianxin Wang, Vishnu Kumarasamy, Yin Wan, Mark D. Long, Michael E. Feigin, Agnieszka K. Witkiewicz, Karen M. Mann, Erik S. Knudsen, Ethan V. Abel

**Affiliations:** 1 Department of Molecular and Cellular Biology, Roswell Park Comprehensive Cancer Center, Elm and Carlton Streets, Buffalo, 14263 NY USA; 2 Department of Pharmacology and Therapeutics, Roswell Park Comprehensive Cancer Center, Buffalo, NY USA; 3 Department of Biostatistics and Bioinformatics, Roswell Park Comprehensive Cancer Center, Buffalo, NY USA; 4 Department of Biostatistics, University at Buffalo, Buffalo, NY USA; 5 Department of Molecular Oncology, Moffitt Cancer Center, Tampa, FL USA; 6 Department of Gastrointestinal Oncology, Moffitt Cancer Center, Tampa, FL USA

**Keywords:** PDAC, Pancreatic ductal adenocarcinoma, HNF1A, FGFR4, Metastasis, FGFR4 inhibitors

## Abstract

**Background:**

We previously identified an oncogenic role for the transcription factor HNF1A in pancreatic ductal adenocarcinoma (PDAC). However, the role of HNF1A in the metastatic progression of PDAC remains unknown and targeting modalities for HNF1A-dependent phenotypes have yet to be identified.

**Methods:**

Transwell chambers were used to assess the effects of HNF1A and FGFR4 modulation on the migration and invasion of ATCC and patient-derived PDAC cells in vitro. An intrasplenic injection xenograft model was used to evaluate the impact of HNF1A knockdown and overexpression on metastatic tumor burden. Single-cell RNA sequencing (scRNA-seq), tissue microarray (TMA) data, and UMAP spatial profiling were used to identify FGFR4 as an HNF1A target gene upregulated in metastatic cells. RNAi and two FGFR4 inhibiting modalities (H3B-6527 and U3-1784) were utilized to demonstrate the efficacy of FGFR4 inhibiting agents at reducing HNF1A-driven metastasis.

**Results:**

Knockdown of HNF1A significantly decreases and HNF1A overexpression significantly increases PDAC cell migration and invasion. In vivo studies show that HNF1A knockdown significantly abrogates metastasis, while overexpression significantly promotes metastasis. scRNA-seq shows that FGFR4 is upregulated in metastatic PDAC cells and staining for HNF1A and FGFR4 in a PDAC TMA reveals significant correlation between HNF1A and FGFR4 in PDAC patients. Further, knockdown and inhibition of FGFR4 significantly decreases HNF1A-mediated cell migration and invasion, and blocks HNF1A-driven metastasis in vivo*.*

**Conclusions:**

These findings demonstrate that HNF1A drives PDAC metastasis via upregulation of FGFR4, and FGFR4 inhibition is a potential mechanism to target metastasis in PDAC patients.

**Supplementary Information:**

The online version contains supplementary material available at 10.1186/s12943-025-02408-5.

## Background

Pancreatic ductal adenocarcinoma (PDAC) is the most common form of pancreatic cancer (~ 90%) and is currently the 3rd leading cause of cancer-related deaths, with a dismal 5-year survival rate of only 13.3% [1]. This poor prognosis is predominantly attributed to metastatic disease, as over 50% of patients present with metastasis, making them ineligible for curative surgery [1]. For these patients, who often succumb to the disease in less than one year, new treatment options are urgently needed. Further demonstrating the aggressiveness of PDAC metastases, approximately 80% of patients with clinically localized PDAC who undergo surgical resection will still die from recurrent metastatic PDAC [2]. As largely ineffective and toxic chemotherapeutics are the only treatment option available to metastatic and relapsed patients, there is a critical and unmet need for a better understanding of the pathways driving PDAC metastasis and identification of actionable drivers of these pathways.

Hepatocyte nuclear factor 1 α (HNF1A) is a gastrointestinal lineage transcription factor that controls the differentiation of liver and pancreatic cells [3–5]. We previously demonstrated an oncogenic role for HNF1A in PDAC. In functional studies, HNF1A overexpression transformed non-tumorigenic pancreatic cells and knockdown of HNF1A significantly depleted tumor growth in a patient-derived xenograft model [6]. Studies in prostate, renal, and colorectal cancers further support an oncogenic function for HNF1A [7–9]. Recent work by Cai et al. found that the HNF1A binding motif is the only transcription factor motif significantly enriched in the super enhancers of metastatic colorectal cancer and PDAC cell lines as compared to primary cell lines, suggesting a role for HNF1A in PDAC metastasis [10]. However, studies investigating the role of HNF1A in metastatic progression are limited.

Fibroblast growth factor receptor 4 (FGFR4) is a transcriptional target gene of HNF1A [6, 11]. FGFR4 is a transmembrane receptor tyrosine kinase (RTK) that is normally expressed in the liver and regulates bile acid synthesis in response to digestion [12, 13]. As with many RTKs, FGFR4 activation by ligand binding triggers phosphorylation and subsequent internal signaling cascades, including the PI3K/Akt and MAPK pathways, to induce cellular responses such as proliferation, differentiation, metabolism, and migration. FGFR4 serves an oncogenic function in several cancer types and promotes metastatic spread in other gastrointestinal cancers [14–19]. Furthermore, FGFR4 is a driving oncogene in a subset of hepatocellular carcinomas in which FGF19, a unique ligand of FGFR4, is amplified. Because of this, several FGFR4-specific inhibitors have been developed and are currently undergoing clinical trials. H3B-6527, an FGFR4-specific tyrosine kinase inhibitor and U3-1784, an FGFR4 blocking antibody, were both utilized in this study [20, 21]. Nevertheless, the function of FGFR4 in the metastatic progression of PDAC has yet to be elucidated.

In the current study, we aimed to determine whether HNF1A serves a role in promoting metastasis of PDAC, and the mechanisms by which it does so. Using in vitro and in vivo models, we identified HNF1A as a key driver of metastasis via transcriptional regulation of its target gene FGFR4. Knockdown of HNF1A significantly depleted metastatic tumors, and HNF1A overexpression significantly increased cell migration and invasion in several PDAC cell lines, which was abrogated by FGFR4 genetic knockdown and pharmacological inhibition. Most importantly, FGFR4 blockade was able to reduce HNF1A-driven metastasis in mice. These results suggest that FGFR4 inhibition is a promising avenue for delaying metastatic spread to extend survival in PDAC patients.

## Methods and materials

### Cell culture

Human pancreatic cancer cell line AsPC-1 (RRID: CVCL_0152) was purchased from ATCC (Manassas, VA). UM5 and UM53 (formerly NY5 and NY53, respectively) cell lines were established as low-passage cells isolated from human patient-derived xenografts as previously described [6]. All cell lines used were maintained in RPMI 1640 media supplemented with 10% FBS, 1% antibiotic–antimycotic, and 100ug/mL gentamicin. Cells were incubated at 37° with 5% CO_2_. Cells were routinely tested for mycoplasma contamination using the MycoScope PCR Detection kit (Gentlantis, San Diego, CA) and authenticated by STR profiling (Roswell Park Comprehensive Cancer Center Genomics Shared Resource).

### Lentiviruses

pENTR/D-TOPO/LacZ and pENTR/D-TOPO/HNF1A were generated as previously described [6]. Human FGFR4 was amplified from UM5 cDNA with primers 5’-CAGGCTCCGCGGCCGCCCCCTTCACCATGCGGCTGCTGCTGGCCCTGTTGG-3’ and 5’-TGGGTCGGCGCGCCCACCCTTTCATGTCTGCACCCCAGACCCGAAGG-3’, digested with SacII and Ascl, and cloned into pENTR/D-TOPO using NEBuilder HiFi DNA Assembly master mix and protocol (New England Biolabs, Ipswich, MA). All cDNAs were shuttled into pLenti6.3/UbC/V5-DEST using LR Clonase II enzyme mix and protocol. miR30-based non-targeting control shRNA (5’- AGCGATCTCGCTTGGGCGAGAGTAAGTAGTGAAGCCACAGATGTACTTACTCTCGCCCAAGCGAGAG. GGCACTCTCGCTTGGGCGAGAGTAAGTACATCTGTGGCTTCACTACTTACTCTCGCCCAAGCGAGAT−3’) and HNF1A targeting shRNA (5’-AGCGAGTCCCTTAGTGACAGTGTCTATAGTGAAGCCACAGATGTATAGACACTGTCACTAAGGGACCGGCAGGTCCCTTAGTGACAGTGTCTATACATCTGTGGCTTCACTATAGACACTGTCACTAAGGGACT-3’) were shuttled into pLentipuro5/TRE3G/V5-DEST, an all-in-one doxycycline inducible lentiviral vector developed using LR Clonase II enzyme mix and protocol. For labeling cells with firefly luciferase, PatGFP-Luc2 was recombined into pLenti0.3/EF/V5-DEST, a modified version of pLenti6.3/UbC/V5-DEST with the human EF-1α promoter instead of the human UbC promoter and no downstream promoter/selective marker cassette, to generate pLenti0.3/EF/GW/PatGFP-Luc2 as previously described [6]. To create the ZsGreen1 FGFR4 enhancer reporter, the multiple cloning site and minimal TA promoter from pTA-Luc (Clontech) was cloned upstream of ZsGreen1(Clontech) into pENTR/D-TOPO (Invitrogen, Waltham, MA) to create pENTR/D-TOPO/MCS-TA-ZsGreen1. The FGFR4 intronic enhancer region was amplified with the primers 5’- TCGATAGGTACCGCCGGCTGGAGCTGGGAGTGAGGCG-3’ and 5’- GAGTCTAGATCTGCCGGCGCGAAGACAGCCGCAGGGAC-3’, digested with KpnI and BglII, and subcloned into pENTR/D-TOPO/MCS-TA-ZsGreen1 to generate the wild-type entry vector. The HNF1A consensus site was mutagenized with the primers 5’- GGCAAATTTGCGCGAAACCGCAGTGCACACAGGGCCTTTTG-3’ and 5’- CGGTTTCGCGCAAATTTGCCCCCTCCACCCCCTGCCGC-3’ to generate the HNF1A binding-defective mutant (underlined section is the HNF1A consensus site). Both the wild-type and mutant reporter cassettes were recombined into pLentineo3/BLOCK-iT-DEST with LR Clonase II (Invitrogen) to generate the reporter lentivirus constructs [6]. The mutant HNF1A virus, pENTR/D-TOPO/HNF1A P291 fsinC, contains a frameshift truncating mutation in the transactivation domain of HNF1A [22]. All viruses were packaged in 293FT cells. Cells were treated with lentivirus-containing conditioned media for 48–72 h and then selected with blasticidin (cDNAs), puromycin (shRNAs), or G418 (ZsGreen1 reporter) for up to two weeks before use in studies. Pooled populations of transduced cells were continuously regenerated to prevent genetic drift.

### Inhibitors

H3B-6527 and U3-1784 for in vitro use were purchased from Selleck Chemicals (Houston, TX) and U3-1784 for in vivo use was purchased from Med Chem Express (Monmouth Junction, NJ). H3B-6527 was used at a concentration of 1 uM for the duration described in each figure. U3-1784 was used at a concentration of 5 ug/mL in vitro for the time indicated in each figure.

### siRNA knockdown

siRNAs were purchased from Dharmacon (Lafayette, CO) for Control (Cat# D-001810–01-20), HNF1A (Cat#M-008215–01-0005), FGFR4 sequence 10 (Cat #D-003134–10-0005), or FGFR4 sequence 11 (Cat #D-003134–11-0005). siRNA was combined with RNAiMax Lipofectamine (Invitrogen) in OptiMEM media and incubated at room temperature for 25 min before transfection. Knockdowns were confirmed via western blot. siRNAs were transfected for the times indicated in each figure legend.

### Transwell assays

Cells were plated at a density of 1 × 10 [5] in 100 uL serum free media in the top chamber of Corning transwell 8 um inserts, or Corning Matrigel coated inserts for invasion assays, with 600 uL complete media plated in the bottom compartment. Cells were then incubated at 37° for 24 (AsPC-1 and UM5) or 6 (UM53) hours for migration assays and 48 (AsPC-1 and UM5) or 24 (UM53) hours for invasion assays. At endpoint, any cells remaining in the top chamber were removed with a cotton swab and cells on the reverse side of the chamber were fixed in 37% formaldehyde for 10 min, dried for 10 min, stained with crystal violet for 10 min, and washed with water. Migrated/invaded cells were then counted. For migration and invasion assays testing FGFR4 inhibition or knockdown, cells were pretreated with drug or siRNA for 48 h. Cells pretreated with drug were plated in serum free media containing drug.

### Western blotting

All lysates were collected and boiled in 1 × Laemmli sample buffer with 2% β-mercaptoethanol for 5 min followed by electrophoresis on 4–20% Mini-PROTEAN TGX precast Tris–Glycine-SDS gels (Bio-Rad, Hercules, CA), transfer to low-fluorescent PVDF (Bio-Rad) and incubated overnight in primary antibodies (1 µg/ml) in 1 × Animal-Free Blocking Solution (Cell Signaling Technology, Danvers, MA) plus 0.1% Tween-20. Blots were incubated in DyLight™ 680 (RRID: AB_614942) or 800-conjugated (RRID: AB_2556616) secondary antibodies in 5% milk in TBS plus 0.1% Tween-20 and 0.005% SDS at room temperature for 1 h and imaged/quantified by an Odyssey® CLx imaging system (Li-Cor, Lincoln, NE; RRID: SCR_014579) and Image Studio Lite software (RRID: SCR_013715). Blots were then cropped in Microsoft PowerPoint for clarity. Antibodies were purchased from Cell Signaling Technology (FGFR4 (RRID: AB_10891199), HNF1A (RRID: AB_2728751), E-cadherin (RRID: AB_2291471), Vimentin (RRID: AB_10695459), Slug (RRID: AB_2239535), Zeb1 (RRID: AB_2935802)), Origene (ZsGreen (RRID: AB_2622267)), and Thermo Fisher Scientific (Actin (RRID: AB_10950489)).

### Intrasplenic injection

NOD/SCID/IL2γR^−/−^ (NSG) mice were bred and maintained at Roswell Park Comprehensive Cancer Center’s animal care facilities. Evenly mixed sex 14–15 week old mice were implanted with 500,000 AsPC-1 tumor cells in a volume of 100 µl of sterile PBS in the spleen of the mice. After a period of 5 min, the spleen was removed, and the splenic vessels cauterized. Bioluminescent imaging was then performed at day 0, and once a week after that until endpoint to monitor tumor burden. For knockdown experiments, mice were put on a diet of doxycycline (2 mg/ml)/5% sucrose water and doxycycline chow (VWR, Radnor, PA) to induce the expression of the non-targeting or HNF1A-targeting shRNAs starting one week prior to inoculation until endpoint. At collection, livers were divided into multiple pieces and fixed at different orientations to assure representative sections when cutting. For antibody experiments, mice were treated twice a week with 25 mg/kg via IP injection. Mice were sacrificed at the specified endpoint or if they exhibited signs of distress or if tumors reached a maximum volume of 2cm [3].

### Orthotopic injection

NOD/SCID/IL2γR^−/−^ (NSG) mice were bred and maintained at Roswell Park Comprehensive Cancer Center’s animal care facilities. Evenly mixed sex 7–8 week old mice were orthotopically implanted with 500,000 AsPC-1 tumor cells in a volume of 50 µl (1:1 volume of cell suspension in growth media and Matrigel) into the pancreas of the mice. Bioluminescent imaging was then performed at day 0, and once a week after that until endpoint to monitor tumor burden. For knockdown experiments, mice were put on doxycycline (2 mg/ml)/5% sucrose water and doxycycline chow (VWR) to induce the expression of the non-targeting or HNF1A-targeting shRNAs. Mice were sacrificed at the specified endpoint or if they exhibited signs of distress or if tumors reached a maximum volume of 2cm [3].

### RNA-sequencing

The sequencing libraries were prepared from 200 ng total RNA purified using the TruSeq Stranded Total RNA kit (Illumina Inc, San Diego, CA). Following manufacturer’s instructions, the first step depleted rRNA from total RNA. After ribosomal depletion, the remaining RNA was purified, fragmented and primed for cDNA synthesis. Fragmented RNA was then reverse transcribed into first strand cDNA using random primers. The next step removed the RNA template and synthesized a replacement strand, incorporating dUTP in place of dTTP to generate ds cDNA. AMPure XP beads (Beckman Coulter, Brea, CA) were used to separate the ds cDNA from the second strand reaction mix resulting in blunt-ended cDNA. A single ‘A’ nucleotide was then added to the 3’ ends of the blunt fragments. Multiple indexing adapters, containing a single ‘T’ nucleotide on the 3’ end of the adapter, were ligated to the ends of the ds cDNA, preparing them for hybridization onto a flow cell. Adapter-ligated libraries were amplified by PCR, purified using Ampure XP beads, and validated for appropriate size on a 4200 TapeStation D1000 Screentape (Agilent Technologies Inc., Santa Clara, CA). The DNA libraries were quantitated using KAPA Biosystems qPCR kit, and were pooled together in an equimolar fashion, following experimental design criteria. Each pool was denatured and diluted to 16 pM for On-Board Cluster Generation and sequencing on a HiSeq2500 sequencer using the appropriate paired-end cluster kit and rapid mode SBS reagents following the manufacturer’s recommended protocol (Illumina Inc.).

### TMA and multispectral staining

Multispectral immunofluorescent (mIF) staining was performed on formalin-fixed paraffin-embedded (FFPE) PDAC tissue microarray (TMA) using the Opal 6-Plex Detection Kit (AKOYA Biosciences, Marlborough, MA). FFPE samples were sectioned at 4 µm thickness and placed on charged slides. The prepared slides were dried at 65 °C for at least 2 h. The dried slides were loaded into a BOND RX^m^ Research Stainer (Leica Biosystems) and deparaffinized with BOND Dewax solution (AR9222, Lecia Biosystems). The slides were treated with the Akoya Biosciences mIF staining process using Opal reagents. The process involved serial applications of the following solutions for each biomarker: ER1 (citrate buffer pH 6, AR996, Leica Biosystems) or ER2 (Tris–EDTA buffer pH9, AR9640, Leica Biosystems) epitope retrieval solutions, blocking buffer (Akoya Biosciences), primary antibody, PowerVision Poly-HRP (Leica Biosystems) secondary antibody, Opal fluorophore (Akoya Biosciences). Spectral DAPI (Akoya Biosciences #FP1490) was manually applied following removal of the samples from the BOND RX^m^. Processed slides were preserved with glass coverslips using ProLong Diamond Antifade Mountant (ThermoFisher Scientific). Antibodies used were purchased from Invitrogen (HNF1A, GT4110 (RRID: AB_2538735)), Santa Cruz Biotechnology (FGFR4, A-10 (RRID: AB_2103663)) and Agilent Dako (AE1/AE3 (RRID: AB_2132885). Slides were imaged on the PhenoImager HT Automated Quantitative Pathology Imaging System (AKOYA Biosciences). Further analysis of the slides was performed using in Form Software v2.6.0 225 (AKOYA Biosciences).

PDAC tumor cases were obtained from the surgical pathology files at Thomas Jefferson University. The TMA contained specimens derived from largely consecutive cases between the years 2002 and 2010 under an Institutional Review Board approved protocol [23, 24]. 220 PDAC patient cores with sufficient live cells (threshold > 100 cells per core) were stained and quantitated. Phenoptr software (RRID: SCR_026104) was used to report the percentage of cells that were positive for each protein stained. For correlation of staining intensities, average whole cell staining intensity was measured for each core. Likely dead cells were excluded from both percent positivity and staining intensity analyses.

### Immunohistochemistry

Anti-HNF1A antibody was purchased from Thermo Fisher Scientific (GT4110), anti-FGFR4 was purchased from Santa Cruz (A-10), and anti-Ki67 was purchased from Abcam (Cat #ab15580; RRID: AB_443209). Sections from FFPE tissues from the above mouse experiments were cut and stained with the above antibodies. Staining intensity was scored using 6 representative regions from each slide and calculated as the percent of positive cells.

### AlamarBlue cell viability assays

After the specified amount of time, cells were incubated in complete RPMI media containing 10% AlamarBlue reagent (Invitrogen) for 30 min- 1 h until a colorimetric change was observed. 100 uL of AlamarBlue containing media from each well was then plated in technical triplicates in a 96-well clear bottom/black-walled plate. Fluorescence was then measured on a BioTek Synergy HTX multi-mode reader (RRID: SCR_019749) on Gen5 3.03 software (RRID: SCR_017317) using 540 nM excitation and 590 nM emission wavelengths. All fluorescence was normalized to their respective control group set to 100% cell viability.

### Colony formation assays

200 cells were plated into 12-well plates incubated at 37° with 5% CO_2_ for 14 days. Media was replenished twice a week. At endpoint, cell viability was measured using AlamarBlue as outlined above followed by fixation with 5% formaldehyde and Crystal Violet staining for representative images. For knockdown studies, cells were transfected with siRNA 72 h prior to plating for colony formation.

### Bioluminescent imaging

Bioluminescent imaging was performed on the IVIS Spectrum imager (RRID: SCR_018621; Roswell Park Comprehensive Cancer Center Translational Imaging Shared Resource). Mice were anesthetized using isoflurane and injected intraperitoneally with 100uL of 15 mg/mL luciferin. After a period of 5 min, mice were imaged from a supine position. Bioluminescence was quantified using the LivingImage software (RRID: SCR_014247).

### Tumorsphere formation

Cells grown under normal 2D conditions were either treated with inhibitors or siRNA for the time points indicated in the legends. Cells were then trysinized, washed, and resuspended as single cells in tumorsphere culture media containing 1% N2 supplement, 2% B27 supplement, 1% antibiotic–antimycotic, 20 ng/mL epidermal growth factor (Gibco, Carlsbad, CA), 20 ng/mL human bFGF-2 (Invitrogen), 10 ng/mL BMP4 (Sigma-Aldrich, St. Louis, MO), 10 ng/mL LIF (Sigma-Aldrich). Cells were plated in 12-well Ultra-Low Attachment Plates (Corning, Corning, NY), 200–1000 cells per well, depending on the cell line. Media with inhibitors or plain media (for knockdowns) was supplemented every 3 days. Tumorspheres were allowed to form for 10–14 days before manually counting.

### Quantitative reverse transcription-PCR (qRT-PCR)

Total RNA was extracted using the Direct-zol RNA Miniprep kit and protocol (Zymo Research, Tustin, CA) and reverse transcribed with High-Capacity RNA-to-cDNA Master Mix (Applied Biosystem). SYBR Green PCR was performed using cDNA and iTaq Universal SYBR Green Supermix (Bio-Rad) on a QuantStudio 7 Pro Real-Time PCR System with QuantStudio Design & Analysis Software 2.8.0 (ThermoFisher Scientific). Conditions used for qPCR were 95 °C for 10 min, followed by 40 cycles of 95 °C for 10 s, 60 °C for 15 s, and 72 °C for 20 s. Primers for FGFR4 were 5’- GGTGACTCCTTGACCTCCA −3’ and 5’- GGGGTAACTGTGCCTATTCG −3’. All quantifications were normalized to an endogenous control *ACTB* (5’-TACCTCATGAAGATCCTCACC-3’ and 5’-TTTCGTGGATGCCACAGGAC-3’). The comparative Ct method (ΔCt) was used to quantitate fold changes in FGFR4 mRNA.

### ChIP-sequencing

A confluent 15 cm culture plate of UM5 cells was used per immunoprecipitation. Cells were fixed with 1% formaldehyde for 10 min. Nuclei were collected and chromatin sheared to 1–10 nucleosomes using SimpleChIP Plus Enzymatic Chromatin IP kit and protocol (Cell Signaling Technology). HNF1A was immunoprecipitated with goat polyclonal antibody (Santa Cruz Biotechnology, RRID: AB_648295). Libraries from HNF1A-immunoprecipitated chromatin and input chromatin was prepared by the University of Michigan Sequencing Core and Sequenced on the Illumina HiSeq 4000 (RRID: SCR_016386) [6]. Enrichment of the FGFR4 enhancer was confirmed by ChIP-PCR using the above quantitative PCR protocol and reagents and primers 5’-GCGGTAAATCAGTAACCCG-3’ and 5’-GGTGGGCAATGAGTAACCAG-3’. Percent Input for immunoprecipitated DNA was calculated using the formula 2% × 2^(Ct 2%Input Sample−Ct IP Sample)^.

### Outgrowth assay

2 × 10 [5] cells were plated in suspension in complete media on non-adherent plates in the presence of H3B-6527 or U3-1784 for 48 h to allow sphere formation. To create a 50:50 collagen-I/Matrigel hydrogel, a solution containing 1.5 mg/ml rat tail collagen-I (R&D Systems), 1X MEM, 1X L-glutamine, 1X GlutaMAX, 6.6 mM NaOH, 0.5625% sodium bicarbonate, 1X Anti-Anti, and 10% FBS was prepared on ice. Collagen-I solution was gently mixed with an equal volume of Matrigel solution on ice. Non-adherent tissue culture plates were coated with an acellular layer of 50:50 hydrogel solution, which was allowed to solidify at 37 °C for 30 min. PDAC spheres were collected by centrifugation and resuspended in 50:50 hydrogel solution with corresponding inhibitors added. Spheres, inhibitors, and hydrogel solution was gently mixed by pipetting and layered on top of the acellular layer and allowed to solidify at 37 °C for 30 min. Media containing was then added on top of the gel, followed by monitoring of sphere outgrowth for 72 h.

### Statistical analysis

Data throughout are expressed as the mean ± SEM. Statistically significant differences between two groups was determined by the two-sided Student t-test for continuous data, while ANOVA was used for comparisons among multiple groups with Tukey’s or Dunnett’s post hoc tests where appropriate, with significance defined as *p* < 0.05. GraphPad Prism 10 (RRID: SCR_002798) was used for these analyses. ns = non-significant, *p < 0.05, **p < 0.01, ***p < 0.001, ****p < 0.0001.

### Study approval

All animal protocols were approved by The Institute Animal Care and Use Committee (IACUC) at Roswell Park Comprehensive Cancer Center. The animal welfare assurance number for this study is A3143-01.

## Results

### HNF1A promotes PDAC cell migration and invasion

**Fig. 1:**
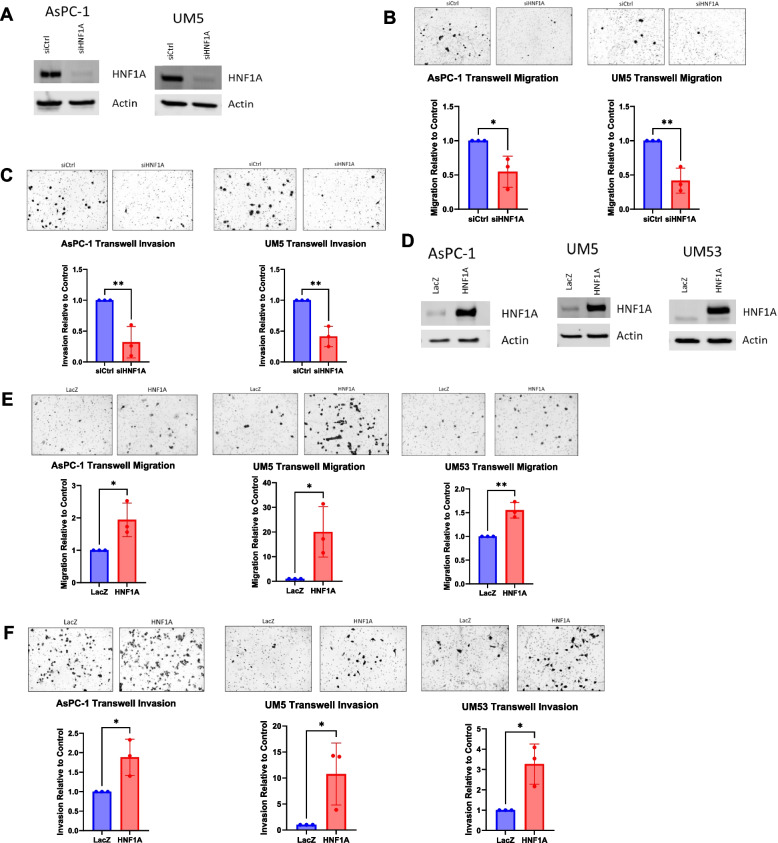
HNF1A promotes PDAC cell migration and invasion. **A** Western blotting for HNF1A in AsPC-1 and UM5 cell lines to confirm knockdown. **B** Normalized quantification and representative images of cell migration with HNF1A knockdown (*n* = 3 biological replicates). Control knockdown or HNF1A knockdown cells were plated in transwell migration chambers and cells that had migrated after 24 h were fixed and stained for counting. **C** Normalized quantification and representative images of cell invasion with HNF1A knockdown (*n* = 3 biological replicates). Control knockdown or HNF1A knockdown cells were plated in transwell invasion chambers and cells that had invaded after 48 h were fixed and stained for counting. **D** Western blotting for HNF1A in AsPC-1, UM5, and UM53 cells lines to confirm overexpression of HNF1A. **E** Normalized quantification and representative images of cell migration with HNF1A overexpression (*n* = 3 biological replicates). LacZ or HNF1A overexpressing cells were plated in transwell migration chambers and cells that had migrated after 6–24 h were fixed and stained for counting. **F** Normalized quantification and representative images of cell invasion with HNF1A overexpression (*n* = 3 biological replicates). LacZ or HNF1A overexpressing cells were plated in transwell invasion chambers and cells that had migrated after 24–48 h were fixed and stained for counting. All bar graphs represent the mean ± SEM and statistical difference was determined by unpaired t-test

Based on its oncogenic role in PDAC [6] and preliminary evidence as a metastatic driver in colorectal cancer [10], we first aimed to establish a pro-metastatic function for HNF1A in PDAC. To determine if HNF1A regulates PDAC metastasis, we first measured cell migration and invasion, as these are key characteristics of metastatic cells. We used transwell chambers to measure cells’ ability to migrate and invade after HNF1A modulation. siRNA mediated knockdown of HNF1A in two PDAC cell lines, AsPC-1 and UM5, which both endogenously express HNF1A (Fig. 1A) resulted in significantly reduced PDAC cell migration through the transwell membrane as compared to cells transfected with a non-targeting control siRNA (Fig. 1B). Cell invasion was measured as a cell’s ability to move through Matrigel coated transwell membranes, which recapitulate the extracellular matrix cells encounter when disseminating from the primary tumor. Consistently, cell invasion was also significantly decreased with HNF1A knockdown (Fig. 1C).

To evaluate whether HNF1A expression is sufficient to drive migration and invasion, we used lentiviral transduction to ectopically overexpress HNF1A in the UM53 cell line, which does not express HNF1A endogenously. We also overexpressed HNF1A in AsPC-1 and UM5 cell lines to evaluate whether the endogenous HNF1A expression fully saturates the migratory and invasive phenotypes or if higher HNF1A expression could further promote the metastatic phenotypes. HNF1A overexpression in all three cell lines (Fig. 1D) significantly increased both cell migration and invasion when compared to the LacZ expressing control cells. This effect was most dramatic in the UM5 cells, with over a ten-fold increase in both migration and invasion with HNF1A overexpression (Fig. 1E and F). Together, these data demonstrate that HNF1A promotes PDAC cell migration and invasion in vitro.

### HNF1A drives PDAC liver metastasisin vivo

Because HNF1A promoted migratory and invasive capabilities, we next sought to determine if HNF1A drives PDAC liver metastasis in vivo. Initial efforts to establish spontaneous metastases with our cell models using orthotopic implantation resulted in exceedingly low penetrance of detectable metastases and circulating tumor cells (Supp Fig. 1).

**Fig. 2:**
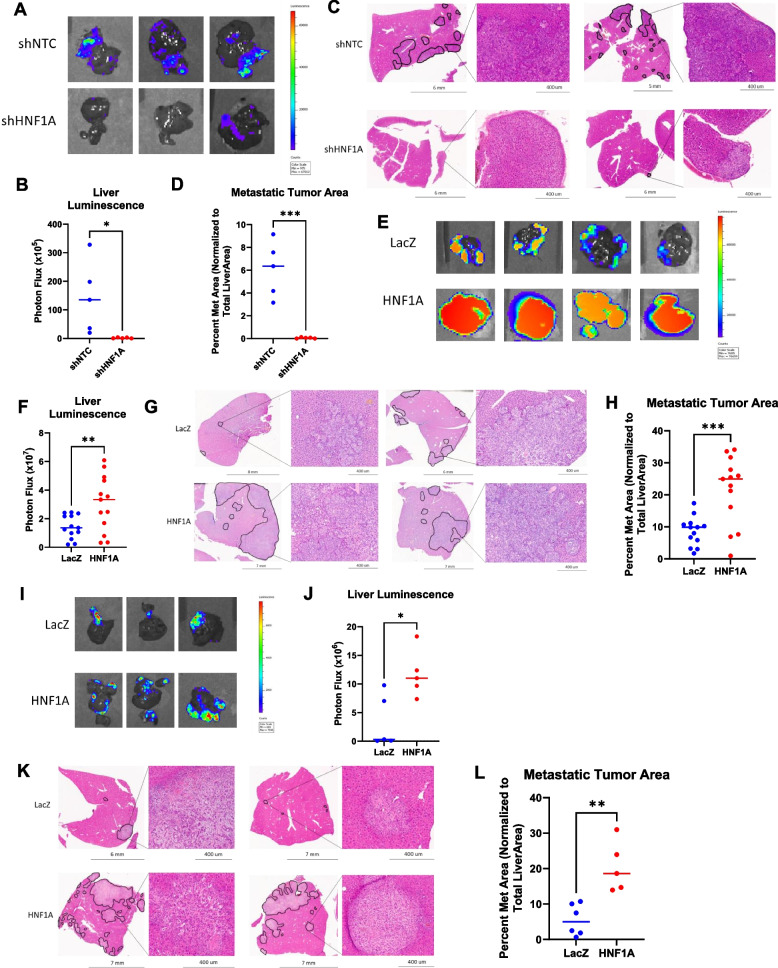
HNF1A drives PDAC liver metastasis in vivo. **A** Representative bioluminescent images of harvested livers from mice implanted with control and HNF1A knockdown cells. **B** Quantification of photon flux from bioluminescent images from all harvested livers at endpoint (*n* = 5 mice per group). **C** Representative images of H&E stained sections of liver tissue from both control and HNF1A knockdown groups. Black outlines indicate metastatic tissue. Zoomed in insets show histology of metastatic lesions. **D** Quantification of total metastatic tumor area normalized as a percentage of total liver area (*n* = 5 mice per group). **E** Representative bioluminescent images of harvested livers from mice implanted with LacZ and HNF1A overexpressing cells. **F** Quantification of photon flux from bioluminescent images from all harvested livers at endpoint (*n* = 13 mice per group). **G** Representative images of H&E stained sections of liver tissue from both LacZ and HNF1A overexpression groups. Black outlines indicate metastatic tissue. Zoomed in insets show histology of metastatic lesions. **H** Quantification of total metastatic tumor area normalized as a percentage of total liver area (*n* = 13 mice per group). **I** Representative bioluminescent images of harvested livers from mice implanted with LacZ and HNF1A overexpressing cells. **J** Quantification of photon flux from bioluminescent images from all harvested livers at endpoint (*n* = 6 mice per group). **K** Representative images of H&E stained sections of liver tissue from both LacZ and HNF1A overexpression groups. Black outlines indicate metastatic tissue. Zoomed in insets show histology of metastatic lesions. **L** Quantification of total metastatic tumor area normalized as a percentage of total liver area (*n* = 6 mice per group). All bar graphs represent the mean and statistical difference was determined by unpaired t-test. *A-H: AsPC-1; I-L: UM53

As such, we then moved on to use the intrasplenic injection liver seeding model of metastasis [25–27], where shNTC or shHNF1A GFP-luciferase tagged AsPC-1 cells were injected intrasplenically into NSG mice (*n* = 5). Bioluminescent imaging was then performed at day 0, then once a week for 4 weeks to assess the ability of the injected cells to initially seed and colonize the liver. At endpoint, mice were sacrificed, and the lung, liver, and pancreas from each mouse were harvested for formalin fixation and paraffin embedment (FFPE). HNF1A depletion with doxycycline administration was confirmed via western blot before inoculation and in tumors via IHC after harvest (Supp Fig. 2A and B). Of note, HNF1A knockdown did not significantly impact cell viability, as both NTC and HNF1A knockdown injected cells produced bioluminescence, which can only be produced by living cells, at the Week 1 timepoint (data not shown). At the four-week endpoint, bioluminescent imaging showed a nearly complete abrogation of liver metastasis with knockdown of HNF1A (Fig. 2A and B). FFPE liver samples were also H&E stained to view histological differences between metastases derived from shNTC or shHNF1A cells. Representative sections of livers were quantified measuring total metastatic tumor area as a percent of the whole liver area. HNF1A knockdown resulted in a near complete elimination of metastatic lesions, with a significant decrease in overall metastatic tumor area from 6.08% in the shNTC livers to 0.06% in the shHNF1A livers (Fig. 2C and D). These data suggest that HNF1A expression is required for liver metastatic colonization.

We next conducted the inverse experiment, in which the same intrasplenic injection was performed with GFP-luciferase tagged AsPC-1 cells overexpressing either LacZ control or HNF1A in NSG mice (*n* = 13 per group), to test whether HNF1A expression can induce PDAC metastasis. HNF1A overexpression was confirmed via western blot before implantation of cells and via IHC after tissue collection (Supp Fig. 2C and D). Bioluminescent imaging was again performed at day 0 and once a week for 6 weeks. Imaging of the harvested livers at the six-week endpoint revealed significantly increased tumor burden in the livers of mice implanted with HNF1A overexpressing cells (Fig. 2E and F). Images of the harvested livers also visibly show a difference in the extent of the metastatic lesions between those seeded with LacZ and HNF1A overexpressing cells (Supp Fig. 2E). The livers from HNF1A overexpressing mice also weighed significantly more than LacZ livers, again indicating increased tumor burden (Supp Fig. 2F). H&E staining of liver sections showed that HNF1A overexpression significantly increased the number and size of metastases, with a median of 8.62% of the total liver area being metastatic tissue in the control mice versus over 21% in the HNF1A livers (Fig. 2G and H). Lung tissues were also collected from mice at endpoint and subjected to H&E staining to assess metastatic tumor development in other organs. While only 3 mice injected with LacZ overexpressing cells developed lung metastases, 8 mice that received HNF1A overexpressing cells developed lung metastases. HNF1A overexpression also resulted in more metastases per lung section (Supp Fig. 2G).

As HNF1A overexpression has previously been shown to increase cell proliferation [6], we further sought to validate that the metastatic tumors from mice inoculated with HNF1A overexpressing cells were not larger simply because they proliferated more rapidly. IHC staining was performed for the proliferative marker Ki67 on the slides from three representative mice from each group and found that the number of Ki67 + cells was not significantly different between LacZ and HNF1A overexpressing tumors (Supp Fig. 2H).

Finally, we sought to determine the effects of introducing HNF1A expression on the intrasplenic model using our UM53 cell line, which does not endogenously express HNF1A. GFP-luciferase tagged UM53 cells transduced with LacZ or HNF1A expression vectors were injected into the spleen of NSG mice (*n* = 6 per group). HNF1A expression was confirmed via western blot before implantation and via IHC in tumors (Supp Fig. 2I and J). Bioluminescent imaging at the 4-week endpoint revealed significantly higher metastatic tumor burden in mice implanted with HNF1A expressing UM53 cells as compared to those inoculated with LacZ cells. FFPE liver sections showed a significant increase in total metastatic tumor area from 5.5% of the total liver area in the LacZ livers to over 20% in the HNF1A livers (Fig. 2K and L). Again, Ki67 staining of liver tumors showed no significant difference in proliferative index, suggesting the difference in tumor size is not confounded by cell proliferation (Supp Fig. 2K). From these data, we concluded that HNF1A expression is necessary and sufficient to promote metastasis.

### HNF1A and FGFR4 expression correlate with human metastatic PDAC

**Fig. 3:**
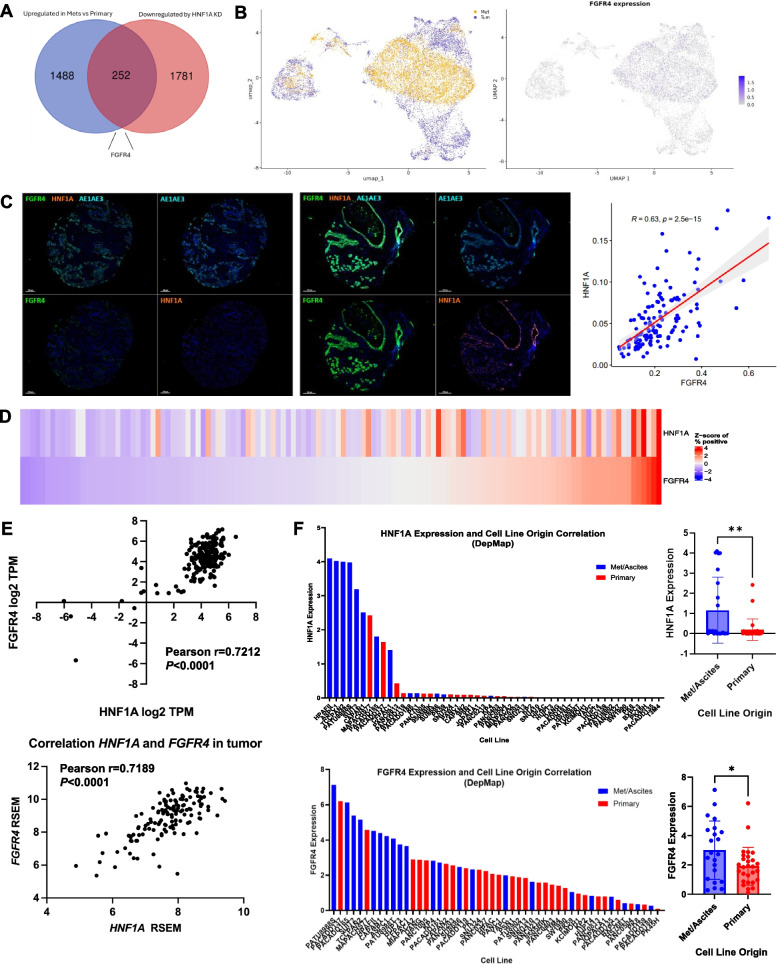
HNF1A and FGFR4 are associated with human metastatic PDAC. **A** Venn diagram comparison of genes upregulated in metastatic tumors vs primary tumors from GSE253260 and genes significantly downregulated with HNF1A knockdown in AsPC-1 cells. **B** Left: UMAP plotting of single cell RNA-sequencing showing spatial grouping of primary (purple) and metastatic (yellow) cells. Right: UMAP plotting FGFR4 expression in all single cells. **C** Left: TMA core demonstrating negativity for both HNF1A and FGFR4 with only staining for pan-cytokeratin. Middle: TMA core showing strong co-staining for all markers. Right: Scatter plot comparing HNF1A and FGFR4 average staining intensity from multispectral immunofluorescent staining of PDAC TMA. **D** Hierarchical organization of staining positivity of individual cores from multispectral immunofluorescent staining of PDAC TMA based on HNF1A and FGFR4 positivity. **E** Correlation of HNF1A and FGFR4 RNA expression in patient tumors from the European Genome Phenome Archive (top) and CPTAC (bottom) datasets. **F** Left: Plotting of all PDAC cell lines available in DepMap Portal based on either HNF1A (top) or FGFR4 (bottom) expression and whether the cell line is derived from primary tumors (red) or metastatic tumors/ascites (blue). Right: Comparison of primary vs metastasis derived cell lines based on the HNF1A (top) or FGFR4 (bottom) expression. Bar graphs represent the mean ± SEM and statistical difference was determined by unpaired t-test

As a transcription factor, HNF1A is currently not a druggable target, limiting its potential as a therapeutic vulnerability. As such, we aimed to identify a targetable downstream mediator of HNF1A-driven metastasis. Taking an unbiased approach, we analyzed publicly available single cell RNA-sequencing data (GSE253260) to assess differentially expressed genes between metastatic tumors and primary tumors from 397 PDAC patients [28]. Of the significantly differentially expressed genes, 1,740 genes were upregulated in metastases vs primary tumors (log2 fold change > 1.5). We then compared this list of upregulated genes to genes significantly downregulated with HNF1A knockdown from our bulk RNA-sequencing in AsPC-1 cells. A substantial number of genes (252) overlapped between these two conditions suggesting that HNF1A may regulate numerous metastasis-associated genes. Of the genes that are both downregulated by HNF1A KD and upregulated in metastases, FGFR4 was a top hit (Fig. 3A). FGFR4 is a known regulator of metastasis in other cancer types and can be therapeutically targeted [16–21, 29–33], though it’s role in PDAC metastasis is uncharacterized. Consistent with a role in PDAC metastasis, spatial FGFR4 expression from the single cell dataset in a UMAP plot showed significant overlap of FGFR4 expression with metastatic tumor cells as compared to primary tumor cells, indicating that FGFR4 expression is higher in metastatic cells as compared to primary tumor cells (Fig. 3B).

To determine if HNF1A and FGFR4 expression are widely associated in PDAC patients, we utilized multispectral immunofluorescence staining of a tumor microarray of 220 PDAC patient samples. These analyses and visual representation revealed that HNF1A and FGFR4 were significantly and positively correlated in patient tumors, with a correlation coefficient of 0.63 (Fig. 3C) (Supp Fig. 3). Hierarchical organization of all TMA samples based on FGFR4 positivity further shows a correlation between HNF1A and FGFR4 expression and also reveals groups of patients that are positive for both HNF1A and FGFR4 or negative for both (Fig. 3D). Using RNA-sequencing datasets from the European Genome Phenome Archive EGAD00001003584 and EGAD00001004548) and CPTAC, we additionally found a positive and significant correlation in HNF1A and FGFR4 RNA levels in patient samples from both primary and metastatic tumors (Fig. 3E) (Supp Fig. 3A) [34–39]. We next sought to assess whether HNF1A and FGFR4 expression are increased in PDAC cells of metastatic origin compared to primary-derived cells. As there is a lack of paired primary/metastatic human PDAC tumor models, we utilized gene expression data from the DepMap Portal containing 22 primary- and 28 metastasis/ascites-derived cell lines [40]. We found that expression of HNF1A and FGFR4 were significantly higher in PDAC cell lines of a metastatic/ascites origin versus primary tumor origin (Fig. 3F). Based on these data, we hypothesized that HNF1A promotes metastasis via transcriptional regulation of FGFR4.

### HNF1A directly regulates FGFR4 expression

**Fig. 4:**
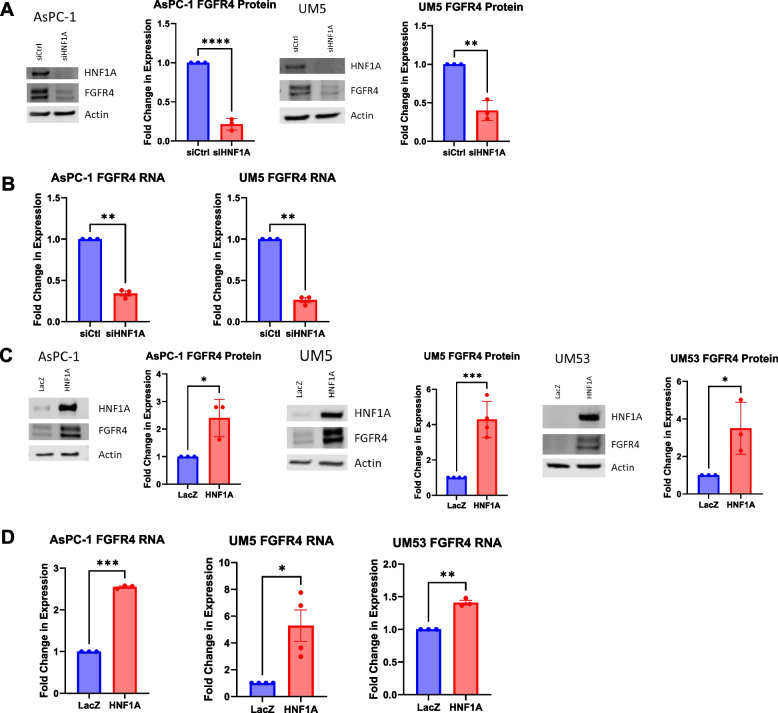
HNF1A regulates FGFR4 expression. **A** Western blotting for HNF1A and FGFR4, and respective quantification of FGFR4 (*n* = 3 biological replicates), with HNF1A knockdown in AsPC-1 and UM5 cell lines. **B** Quantitative RT-PCR analysis of FGFR4 RNA levels 72 h after HNF1A knockdown in AsPC-1 and UM5 cell lines (*n* = 3 biological replicates). **C** Western blotting for HNF1A and FGFR4, and respective quantification of FGFR4 (*n* = 3 biological replicates), with HNF1A overexpression in AsPC-1, UM5, and UM53 cell lines. **D** Quantitative RT-PCR analysis of FGFR4 RNA levels with HNF1A overexpression in AsPC-1, UM5, and UM53 cell lines (*n* = 3 biological replicates). All bar graphs represent the mean ± SEM and statistical difference was determined by unpaired t-test

Based on the strong correlation between HNF1A and FGFR4 expression in metastatic disease, we next sought to validate FGFR4 as a direct target gene of HNF1A. Previous ChIP-sequencing from the ENCODE project in HepG2 cells, a liver cancer cell line that expresses HNF1A, and from our group in UM5 cells found an HNF1A binding peak, which contains the HNF1A binding sequence, in the FGFR4 enhancer region found in the first intron of the *FGFR4* locus (Supp Fig. 3B) [6, 11, 41]. This HNF1A binding peak also overlaps with an H3K27 acetylation peak, indicating an active enhancer. This putative enhancer was previously shown to interact with HNF1A in PDAC cells in vitro, with ectopic expression of HNF1A being able to induce FGFR4 in HNF1A-negative/FGFR4-negative Panc-1 cells [11]. As such, we sought to determine if endogenous HNF1A in fact regulates FGFR4 expression in PDAC cells. We found that siRNA-mediated knockdown of HNF1A reduced FGFR4 protein and RNA expression by more than 50% in both AsPC-1 and UM5 cells lines (Fig. 4A and B). Lentiviral overexpression of HNF1A increased FGFR4 protein and RNA expression in all cell lines, including AsPC-1 and UM5 which endogenously express both HNF1A and FGFR4, demonstrating that HNF1A overexpression can further promote FGFR4 expression (Fig. 4C and D).

**Fig. 5:**
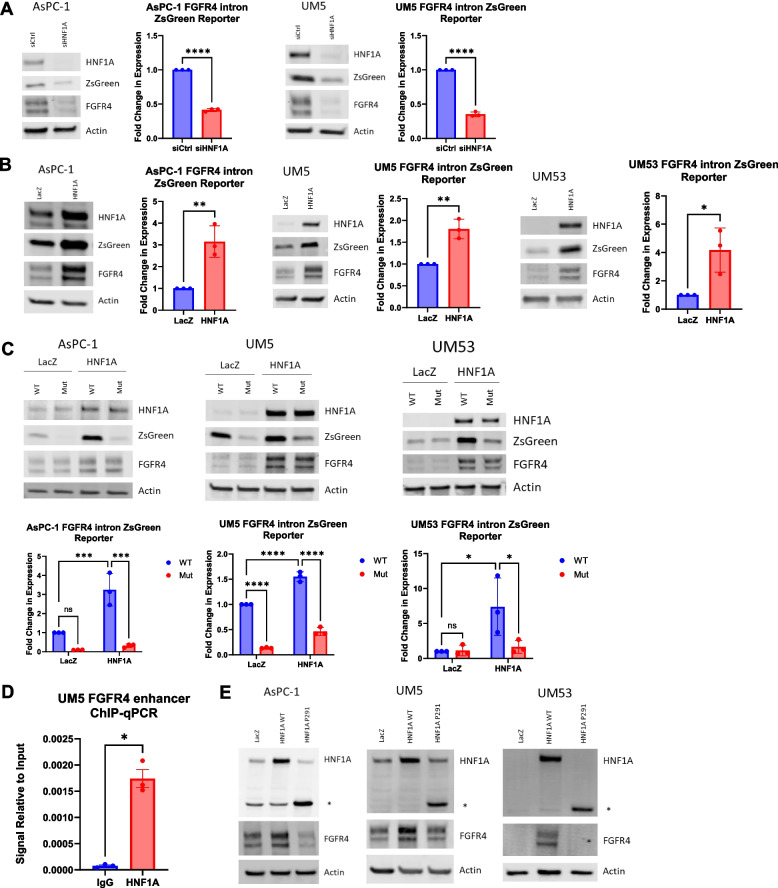
HNF1A directly binds to the FGFR4 enhancer. **A** Western blotting for HNF1A, FGFR4, and ZsGreen1 expression, and respective quantification of ZsGreen1 (*n* = 3 biological replicates), in a reporter system with ZsGreen1 under the control of the FGFR4 enhancer with HNF1A knockdown in AsPC-1 and UM5 cell lines. **B** Western blotting for HNF1A, FGFR4, and ZsGreen1 expression, and respective quantification of ZsGreen1 (*n* = 3 biological replicates), in a reporter system with ZsGreen1 under the control of the FGFR4 enhancer with HNF1A overexpression in AsPC-1, UM5, and UM53 cell lines. **C** Western blotting of HNF1A, FGFR4, and ZsGreen1, and respective quantification of ZsGreen1 (*n* = 3 biological replicates), in the above reporter system with HNF1A overexpression ± mutation of the HNF1A binding motif in the FGFR4 enhancer in AsPC-1, UM5, and UM53 cell lines. **D** ChIP-PCR was performed on UM5 cells using normal IgG or BRD4 antibody. **E** Western blotting for HNF1A and FGFR4 with overexpression of wild-type (WT) HNF1A and HNF1A with a mutant and inactive transactivation domain (P291). All bar graphs represent the mean ± SEM. Statistical difference was determined by unpaired t-test when comparing 2 conditions and two-way ANOVA with Tukey’s multiple comparisons test when comparing 4 conditions

To confirm direct regulation of the *FGFR4* enhancer region by HNF1A, we next designed a reporter in which ZsGreen1 expression is under the control of the *FGFR4* enhancer sequence, which contains the HNF1A consensus sequence. Using this system, we found that HNF1A knockdown significantly depleted ZsGreen1 expression and that HNF1A overexpression significantly induced expression as compared to LacZ overexpressing control cells (Fig. 5A and B). Additionally, we mutated the HNF1A binding sequence in our reporter system, blocking the ability of HNF1A to bind the FGFR4 enhancer region. Mutation of this site resulted in a significant decrease in the HNF1A-induced ZsGreen1 expression (Fig. 5C). Further validating direct binding by HNF1A, ChIP-qPCR of the *FGFR4* enhancer region in UM5 cells showed a significant enrichment for HNF1A binding as compared to IgG control (Fig. 5D). Lastly, western blotting and qRT-PCR for FGFR4 showed that mutant HNF1A with a truncating mutation in the transactivation domain of HNF1A (P291 fsinC) abrogates the ability of HNF1A to upregulate FGFR4 at the protein and RNA level, suggesting that HNF1A transactivation and binding is required for FGFR4 expression (Fig. 5E) (Supp Fig. 3C). Overall, we conclude that HNF1A directly binds an FGFR4 enhancer, promoting FGFR4 expression in PDAC.

### FGFR4 promotes pro-metastatic properties downstream of HNF1A

**Fig. 6:**
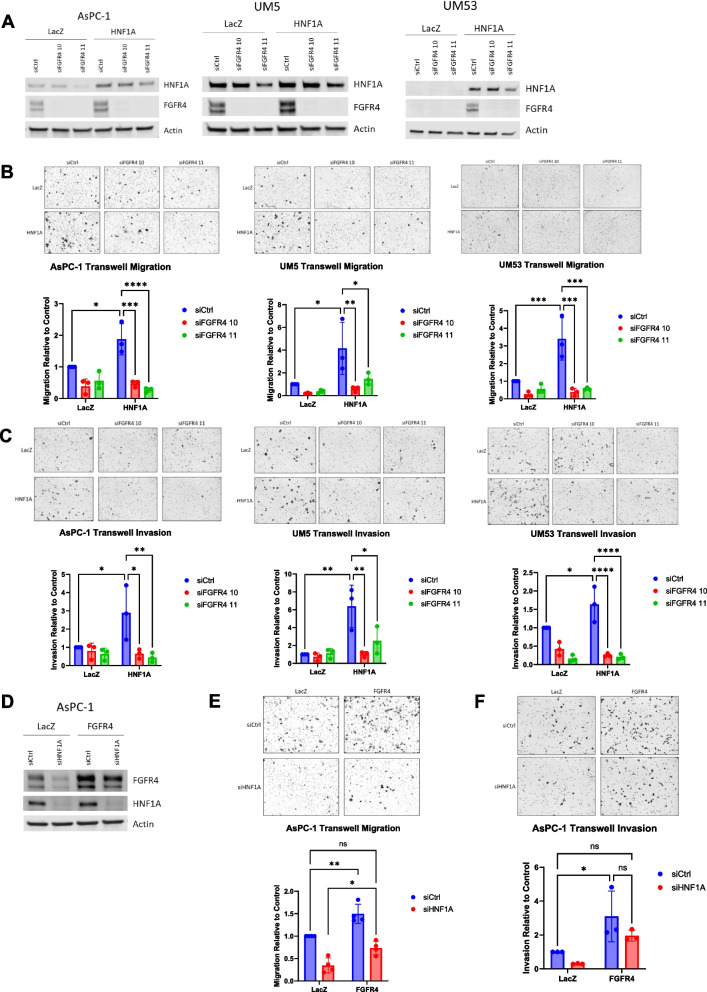
FGFR4 promotes migration and invasion downstream of HNF1A. **A** Western blotting for HNF1A and FGFR4 to confirm HNF1A overexpression and FGFR4 knockdown in AsPC-1, UM5, and UM53 cell lines. **B** Normalized quantification and representative images of cell migration in both LacZ and HNF1A overexpressing sublines with FGFR4 knockdown (*n* = 3 biological replicates). Control or FGFR4 knockdown cells were plated in transwell migration chambers and cells that had migrated after 6–24 h were fixed and stained for counting. **C** Normalized quantification and representative images of cell invasion in both LacZ and HNF1A overexpressing sublines with FGFR4 knockdown (*n* = 3 biological replicates). Control or FGFR4 knockdown cells were plated in transwell invasion chambers and cells that had migrated after 24–48 h were fixed and stained for counting. **D** Western blotting for HNF1A and FGFR4 to confirm FGFR4 overexpression and HNF1A knockdown in AsPC-1 cells. **E** Normalized quantification and representative images of cell migration in both LacZ and FGFR4 overexpressing sublines with HNF1A knockdown (*n* = 4 biological replicates). Control or FGFR4 knockdown cells were plated in transwell migration chambers and cells that had migrated after 24 h were fixed and stained for counting. **F** Normalized quantification and representative images of cell invasion in both LacZ and FGFR4 overexpressing sublines with HNF1A knockdown (*n* = 3 biological replicates). Control or FGFR4 knockdown cells were plated in transwell invasion chambers and cells that had migrated after 48 h were fixed and stained for counting. All bar graphs represent the mean ± SEM and statistical difference was determined by two-way ANOVA with Tukey’s multiple comparisons test

We next aimed to ascertain whether FGFR4, as a direct target gene of HNF1A, is responsible for the HNF1A-mediated metastatic phenotype. To evaluate this, we performed FGFR4 knockdown using two different siRNA sequences in both the LacZ and HNF1A overexpressing sublines of AsPC-1, UM5, and UM53 (Fig. 6A). These cell line conditions were then plated for transwell migration and invasion assays, and we found that depletion of FGFR4 expression significantly reduced both the migration and invasion induced by HNF1A expression either back to or even below the LacZ siCtrl condition in all three cell lines (Fig. 6B and C). Importantly, these effects were not due to a growth differential caused by knockdown of FGFR4. The two siRNA sequences did not significantly affect cell viability, colony formation, tumorsphere formation, or EMT marker expression, except for significant decreases in colony formation in UM53 cells and tumorsphere formation in UM5 cells (Supp Fig. 4A-D).

To ascertain whether FGFR4 is sufficient to promote metastatic properties, we also tested whether FGFR4 overexpression could rescue cell migration and invasion from the decrease induced by HNF1A knockdown. FGFR4 overexpression and HNF1A knockdown in AsPC-1 cells (Fig. 6D) revealed that FGFR4 overexpression alone significantly increased cell migration and invasion as compared to LacZ control cells. More importantly, overexpression of FGFR4 was able to rescue migration and invasion from the effects of HNF1A knockdown, as cells with HNF1A knockdown and FGFR4 overexpression migrated and invaded significantly more than those with HNF1A knockdown alone (Fig. 6E and F). Again, the effects of FGFR4 are specific to migration and invasion as cell viability, colony formation, tumorsphere formation, and EMT marker expression were not significantly changed with FGFR4 overexpression, except for colony formation in UM5 cells (Supp Fig. 4E-H). These data strongly support the hypothesis that FGFR4 is responsible for HNF1A-driven PDAC cell migration and invasion.

### Pharmacologic inhibition of FGFR4 ablates HNF1A-driven metastasis

We further wanted to elucidate whether pharmacologic inhibition of FGFR4 could recapitulate the effects of FGFR4 knockdown. FGFR4 targeting agents are an area of high interest in liver cancer, where a subset of patients exhibits an amplification of FGF19, a unique FGFR4 ligand. Because of this, several classes of FGFR4 inhibitors have been developed and tested in clinical trials. To test the effects of pharmacologic blockade of FGFR4 on HNF1A-driven metastatic phenotypes, we used two FGFR4 inhibiting modalities; H3B-6527, an FGFR4-specific tyrosine kinase inhibitor [20], and U3-1784, an FGFR4 blocking antibody [21].

**Fig. 7:**
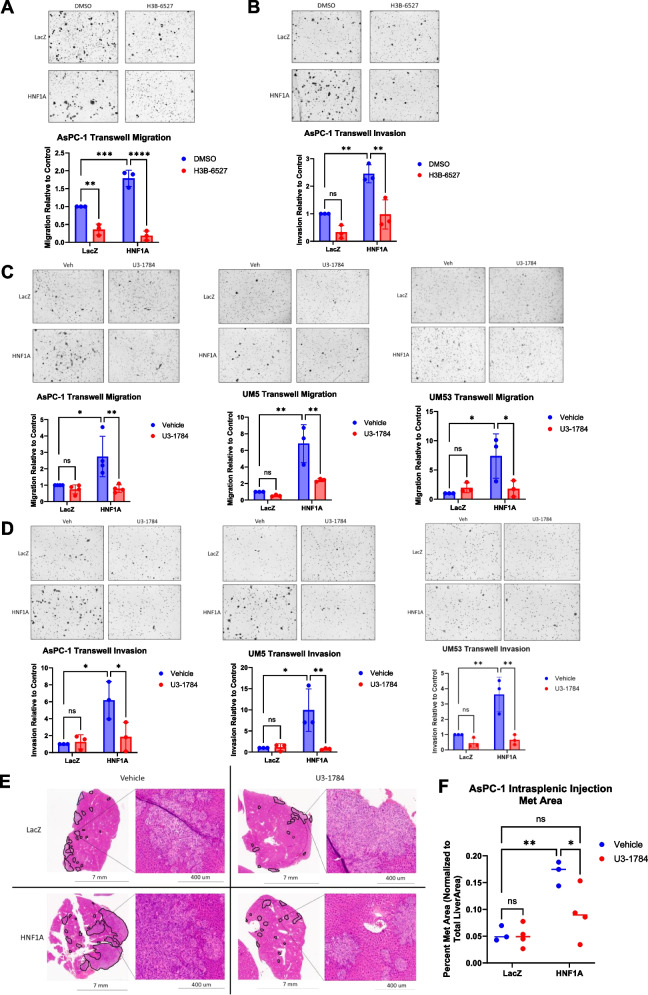
Pharmacologic inhibition of FGFR4 reduces HNF1A-driven metastasis. **A** Normalized quantification and representative images of cell migration in both LacZ and HNF1A overexpressing sublines of AsPC-1 cells treated with 1 μM H3B-6527 (*n* = 3 biological replicates). DMSO or H3B-6527 treated cells were plated in transwell migration chambers in serum free media containing DMSO or H3B-6527 and cells that had migrated after 24 h were fixed and stained for counting. **B** Normalized quantification and representative images of cell invasion in both LacZ and HNF1A overexpressing sublines of AsPC-1 cells treated with 1 μM H3B-6527 (*n* = 3 biological replicates). DMSO or H3B-6527 treated cells were plated in transwell invasion chambers in serum free media containing DMSO or H3B-6527 and cells that had migrated after 48 h were fixed and stained for counting. **C** Normalized quantification and representative images of cell migration in both LacZ and HNF1A overexpressing sublines of AsPC-1, UM5, and UM53 cells treated with 5 μg/mL U3-1784 (*n* = 4 biological replicates). Vehicle or U3-1784 treated cells were plated in transwell migration chambers in serum free media containing vehicle or U3-1784 and cells that had migrated after 24 h were fixed and stained for counting. **D** Normalized quantification and representative images of cell invasion in both LacZ and HNF1A overexpressing sublines of AsPC-1, UM5, and UM53 cells treated with 5 μg/mL U3-1784 (*n* = 3 biological replicates). Vehicle or U3-1784 treated cells were plated in transwell invasion chambers in serum free media containing vehicle or U3-1784 and cells that had migrated after 48 h were fixed and stained for counting. **E** Representative images of H&E stained sections of liver tissue from both LacZ and HNF1A groups with either vehicle or U3-1784 treatment. Black outlines indicate metastatic tissue. Zoomed in insets show histology of metastatic lesions. **F** Quantification of total metastatic tumor area normalized as a percentage of total liver area (*n* = 3 mice per group for vehicle treated, 4 mice per group for U3-1784 treated). All bar graphs represent the mean ± SEM and statistical difference was determined by two-way ANOVA with Tukey’s multiple comparisons test

Consistent with the genetic manipulation findings, we found that treatment with H3B-6527 significantly decreased cell migration and invasion promoted by HNF1A overexpression in AsPC-1, UM5, and UM53 cells (Fig. 7A and B) (Supp Fig. 7A and B). Similarly, U3-1784 treatment was able to significantly reduce HNF1A-induced migration and invasion back to or below LacZ Vehicle treated baseline levels in all cell lines (Fig. 7C and D). Both agents also significantly reduced 3D outgrowth of HNF1A-overexpressing but not LacZ-overexpressing UM53 cells in collagen I/Matrigel hybrid hydrogels (Supp Fig. 7C), further demonstrating that inhibition of FGFR4 abrogates HNF1A-dependent invasion. Treatment with these agents did not perturb total FGFR4 expression (Supp Fig. 5). These results were not due to any effect on cell viability by the FGFR4 inhibitors, as cell viability and colony formation were only minimally impacted by treatment with either H3B-6527 or U3-1784 (Supp Fig. 6A-B, D-E). Furthermore, treatment with the inhibitors did not affect other metastasis-related phenotypes such as stemness or EMT, as tumorsphere formation and EMT marker expression remained unchanged with treatment (Supp Fig. 6 C, F, and G).

Finally, to assess the efficacy of FGFR4 inhibition on reducing metastasis in vivo, we treated mice with either vehicle control or 25 mg/kg of U3-1784 twice a week for 4 weeks following intrasplenic injection of either LacZ or HNF1A overexpressing sublines of GFP-luciferase tagged AsPC-1 cells. Livers from these mice were harvested at endpoint and the FFPE tissues were H&E stained (Fig. 6E). As before, total metastatic tumor area was measured and normalized to total liver area. Expectedly, the livers from mice inoculated with HNF1A overexpressing cells had significantly increased metastatic tumor burden as compared to mice implanted with LacZ cells. However, treatment with the FGFR4 blocking agent significantly reduced the development and growth of metastatic lesions in the HNF1A livers (Fig. 6F). Importantly, HNF1A and FGFR4 protein levels were unchanged by treatment as assessed via IHC on liver sections (Supp Fig. 7D). These changes in metastatic tumor burden were not attributed to proliferation, as Ki67 positivity was not significantly different between groups (Supp Fig. 7E). Overall, these findings strongly indicate that treatment with an FGFR4 inhibitor is a clinically viable avenue for the disruption of HNF1A-driven metastasis in PDAC.

## Discussion

In the current study, we identified the transcription factor HNF1A as a driver of metastasis in PDAC, introducing a novel pathway in the progression of pancreatic cancer. Functional data reveal that loss of HNF1A dramatically reduces cell migration and invasion in vitro and nearly eliminates PDAC liver metastasis in vivo. Our data further show that the receptor tyrosine kinase FGFR4 is a therapeutic vulnerability in this signaling axis. Knockdown or inhibition of FGFR4, using two different inhibitory agents, significantly reduced the HNF1A-mediated increase in migration, invasion, and metastasis.

One of the major takeaways from this work is the clinical applicability of FGFR4 inhibitors for the treatment of PDAC, and there are several treatment scenarios in which FGFR4 inhibiting agents are likely to be efficacious for PDAC patients. The first is as a neoadjuvant therapy for patients with resectable or borderline resectable primary tumors, categories which approximately 50% of PDAC patients fall into. Because the large majority of patients that undergo surgery will succumb to metastatic relapse [1, 2], FGFR4 inhibition before surgery could improve the potential for cure with resection by preventing recurrent metastatic tumors. Second, a small subpopulation of the public is regularly screened for pancreatic cancer due to their family history and/or risk factors. Similar to neoadjuvant treatment for surgery, treatment with FGFR4 inhibitors at the earliest signs of disease for these patients could prevent the characteristically early metastatic spread of PDAC, leading to an improved survival outcome. Unfortunately, most PDAC patients present with distant metastases already present, leaving them with a bleak prognosis. Though FGFR4 inhibitors are unlikely to deplete existing metastatic tumors as they are not cytotoxic, preventing or delaying further lesions could allow standard of care chemotherapies to prove more efficacious and extend survival. Lastly, the work done in this study provides evidence that HNF1A and FGFR4 could serve as biomarkers for treatment stratification, as patients with HNF1A^high^ and/or FGFR4^high^ tumors will likely respond well to FGFR4 inhibitors. FGFR4 expression may also serve as a biomarker for response to erlotinib, while not a direct target itself, as recent work by Rao et al*.* showed that patients with HNF1A positivity had significantly better survival as compared to HNF1A negative patients when treated with gemcitabine plus erlotinib [42]. Collectively, FGFR4 and responsiveness to erlotinib may indicate that HNF1A regulates a larger RTK network, which should be explored more extensively in future studies.

Other groups have previously proposed tumor suppressive functions for both HNF1A and FGFR4 [43, 44]. However, in addition to our current findings demonstrating an oncogenic and pro-metastatic role for HNF1A, our group has previously published that HNF1A is an oncogene by promoting stem-like properties in PDAC cells [6]. An oncogenic role for HNF1A in PDAC and other cancers has been supported by other studies as well [7, 9]. Regarding FGFR4, one key divergence in our work from D’Agosto et al. [43], who show a tumor suppressive role for FGFR4 in PDAC, is the selection of model systems and FGFR4 inhibitor. Our ability to replicate our findings across several cell lines, including primary PDAC cells, lends to the rigor of our results. We also observed consistent results using multiple modalities of FGFR4 modulation and inhibition, including both a tyrosine kinase inhibitor and blocking antibody, and the FGFR4 inhibiting therapies selected for use in this study are more clinically relevant, as they have been tested in humans. By contrast, the first-generation FGFR4 BLU-9931 used by D’Agosto et al*.* never advanced to clinical trials due to the toxicity profile observed in mice, potentially due to off-target activity [43, 45]. These off-target effects may therefore be the cause of the discrepancies seen between their findings and ours. Though there is no clear reason for the discordance with the results of D’Agosto et al*.*, we conducted cell viability, proliferation, and stemness studies with FGFR4 knockdown and inhibition and found no significant impact to these phenotypes, and even found a trend towards FGFR4 being a positive regulator of cell proliferation. This supports a pro-metastatic role for FGFR4 that is independent of other malignant programs in PDAC.

An encouraging finding of our work is the lack of cytotoxic effects with FGFR4 depletion or inhibition, lending to the study’s translatability. Unlike pan-FGFR inhibitors, or even some FGFR1-3 specific inhibitors, the two FGFR4 targeting modalities used in this work showed minimal effects on cell viability and colony formation, suggesting a lack of harsh toxicities with these therapies. Moreso, treatment with U3-1784 in vivo did not lead to any significant changes in body weight as compared to the vehicle treated groups, again indicating that this regimen is likely tolerable. Clinical trial data of other FGFR4 inhibitors found that one of the most common side effects of these agents is increased bile acid production, though this could be offset with the addition of a bile acid sequestrant [29, 33, 46]. Therefore, FGFR4 inhibition is a promising avenue for the safe and effective treatment of PDAC.

We acknowledge that a limitation of this work is the absence of a biomarker for the on-target effects of FGFR4 inhibition. Knockdown or inhibition of FGFR4 did not result in a change in any of the canonical FGFR4 downstream targets, including the PI3K/Akt and MAPK pathways, or in any metastasis-associated programs such as EMT or stemness. Effects on migration, invasion, and metastasis served as functional readouts of FGFR4 loss, though a molecular biomarker was not found. Future work will therefore need to address this to identify a readout of the efficacy of the FGFR4 inhibitors used in this study, which will not only establish biomarkers of on-target inhibition but will also identify other potential vulnerabilities. One interesting finding from this study is the lack of effect of FGFR4 knockdown or inhibition in the LacZ context, even though these cells still express FGFR4. Though not tested in this study, it is possible that this is because of other HNF1A targets that may be involved in metastatic progression, independent of FGFR4. The comparison of HNF1A regulated genes and metastasis associated genes revealed that over 200 putative HNF1A target genes are correlated with metastasis. This suggests that there may be other players involved in HNF1A’s regulation of metastatic spread in PDAC and that these players are able to compensate for the loss of FGFR4 signaling. Direct inhibition of HNF1A expression and/or activity, such as via PROTACs, may therefore be a promising channel to overcome this challenge by blocking all HNF1A metastasis-promoting pathways.

In summary, we identified a novel role for HNF1A in the metastatic progression of PDAC through its transcriptional regulation of FGFR4. This therapeutic vulnerability may, in turn, be leveraged by use of FGFR4 inhibitors to block or delay the spread of PDAC to vital organs and extend patient survival.

## Conclusions

Pancreatic ductal adenocarcinoma (PDAC) is one of the most lethal malignancies, made even more devastating when metastases overwhelm major organs. The vast majority of PDAC patients either present with metastases or will relapse with recurrent metastatic PDAC after primary tumor resection. Unfortunately, toxic and largely ineffective chemotherapies are currently the only approved treatment options for these patients and therefore there exists a critical and unmet clinical need for targeted therapies against pro-metastatic pathways in PDAC. In the current study, we identify HNF1A as an oncogenic transcription factor that drives metastasis in PDAC, and it does so through upregulation of the receptor tyrosine kinase FGFR4. Importantly, FGFR4 is a targetable vulnerability and treatment with an FGFR4 blocking antibody reduces HNF1A-driven metastasis. These findings suggest that FGFR4 inhibitors could be an efficacious treatment for PDAC patients for the prevention or delay of metastatic tumor development, leading to improved patient outcomes.

## Supplementary Information

Supplementary Material 1: Supp Fig. 1. A) Quantification of photon flux from bioluminescent images from all harvested livers and lungs inoculated with LacZ or HNF1A overexpressing cells at endpoint (*n* = 10 mice per group). B) Quantification of photon flux from bioluminescent images from all harvested livers and lungs with control or HNF1A knockdown at endpoint (*n* = 10 mice per group). C) Percent of blood cells positive for GFP (tumor cells) collected from the cardiac blood. All bar graphs represent the mean and statistical difference was determined by unpaired t-test. Supp Fig. 2. A) Western blotting for HNF1A with and without doxycycline administration before cell inoculation. B) Immunohistochemistry for HNF1A in the liver metastases of mice from each respective arm. C) Western blotting for HNF1A in AsPC-1 cells before inoculation. D) Immunohistochemistry for HNF1A in the liver metastases of mice from each respective arm. E) Representative photographs of harvested livers from both LacZ and HNF1A groups showing increased tumor burden (white tissue) in the HNF1A livers. F) Measured liver weights of all livers after harvest at endpoint (*n* = 13 mice per group). G) Quantification of the number of lung metastases per H&E stained tissue section. H) Quantification of cells positive for Ki67 immunohistochemistry staining in the liver metastases from 3 representative mice from each group. I) Western blotting for HNF1A in UM53 cells before inoculation. J) Immunohistochemistry for HNF1A in the liver metastases of mice from each respective arm. K) Quantification of cells positive for Ki67 immunohistochemistry staining in the liver metastases from 3 representative mice from each group. All bar graphs represent the mean and statistical difference was determined by unpaired t-test. Supp Fig. 3. A) Correlation of HNF1A and FGFR4 RNA expression in patient liver metastatic tumors from the European Genome Phenome Archive dataset. B) ChIP-sequencing of HNF1A and H3K27ac reads from HepG2 (top) and HNF1A reads from UM5 (bottom) cells. C) Quantitative RT-PCR analysis of FGFR4 RNA levels with overexpression of wild-type (WT) HNF1A and HNF1A with a mutant and inactive transactivation domain (P291) (*n* = 3 biological replicates). All bar graphs represent the mean ± SEM and statistical difference was determined by two-way ANOVA with Tukey’s multiple comparisons test or one-way ANOVA with Tukey’s multiple comparisons test. Supp Fig. 4. A) Cell viability as normalized to control knockdown 72 h after siRNA-mediated knockdown of FGFR4. B) Cell viability as normalized to control knockdown for colony formation experiments. Cells were transfected with siRNA for 72 h before plating at a low cell density (200 cells/well) and grown for 2 weeks. Resultant colonies were quantified by AlamarBlue viability assay. C) Number of tumorspheres formed after 14 days with siRNA-mediated knockdown of FGFR4. D) Western blotting for HNF1A, FGFR4, E-cadherin, Zeb1, Vimentin, Slug, and Actin 72 h after siRNA-mediated knockdown of FGFR4. E) Cell viability as normalized to LacZ control overexpression 72 h after plating. F) Cell viability as normalized to LacZ control overexpression for colony formation experiments. Cells were plated at a low cell density (200 cells/well) and grown for 2 weeks. Resultant colonies were quantified by AlamarBlue viability assay. G) Number of tumorspheres formed after 14 days with overexpression of FGFR4. H) Western blotting for HNF1A, FGFR4, E-cadherin, Zeb1, Vimentin, Slug, and Actin with overexpression of FGFR4. All bar graphs represent the mean and statistical difference was determined by unpaired t-test. Supp Fig. 5. A) Western blotting for HNF1A and total FGFR4 in AsPC-1, UM5, and UM53 cell lines treated with either DMSO or H3B-6527. B) Western blotting for HNF1A and total FGFR4 in AsPC-1, UM5, and UM53 cell lines treated with either Vehicle or U3-1784. Supp Fig. 6. A) Cell viability of LacZ and HNF1A overexpressing AsPC-1, UM5, and UM53 cells treated with DMSO or H3B-6527 as normalized to LacZ control cells treated with DMSO after 72-h treatment. B) Cell viability for colony formation experiments as normalized to LacZ cells treated with DMSO. Cells were treated with wither DMSO or H3B-6527 for 72 h before plating at a low cell density (200 cells/well) and grown for 2 weeks. Resultant colonies were quantified by AlamarBlue viability assay. C) Number of tumorspheres formed after 14 days with continuous treatment with 1 μM H3B-6527. D) Cell viability of LacZ and HNF1A overexpressing AsPC-1, UM5, and UM53 cells treated with Vehicle or U3-1784 as normalized to LacZ control cells treated with DMSO after 72-h treatment. E) Cell viability for colony formation experiments as normalized to LacZ cells treated with DMSO. Cells were treated with wither DMSO or H3B-6527 for 72 h before plating at a low cell density (200 cells/well) and grown for 2 weeks. Resultant colonies were quantified by AlamarBlue viability assay. F) Number of tumorspheres formed after 14 days with continuous treatment with 5 μg/mL U3-1784. G) Western blotting for HNF1A, FGFR4, E-cadherin, Zeb1, Vimentin, Slug, and Actin 72 h after treatment with H3B-6527 or U3-1784. All bar graphs represent the mean and statistical difference was determined by two-way ANOVA with Tukey’s multiple comparisons test. Supp Fig. 7. A) Normalized quantification and representative images of cell migration in both LacZ and HNF1A overexpressing sublines of UM5 and UM53 cells treated with 1 μM H3B-6527(*n* = 3 biological replicates). DMSO or H3B-6527 treated cells were plated in transwell migration chambers in serum free media containing DMSO or H3B-6527 and cells that had migrated after 24 h were fixed and stained for counting. B) Normalized quantification and representative images of cell invasion in both LacZ and HNF1A overexpressing sublines of UM5 and UM53 cells treated with 1 μM H3B-6527 (*n* = 3 biological replicates). DMSO or H3B-6527 treated cells were plated in transwell invasion chambers in serum free media containing DMSO or H3B-6527 and cells that had migrated after 48 h were fixed and stained for counting. C) Cell invasion as normalized to DMSO treated controls for UM53 spheres seeded in a collagen/Matrigel matrix at 0, 24, and 48 h treated with either H3B-6527 or U3-1784. D) Immunohistochemistry for HNF1A (left) and FGFR4 (right) in the liver metastases of mice from each respective arm. E) Quantification of cells positive for Ki67 immunohistochemistry staining in the liver metastases from 3 representative mice from each group. All bar graphs represent the mean ± SEM and statistical difference was determined by two-way ANOVA with Tukey’s multiple comparisons test.

## Data Availability

The single cell RNA-sequencing datasets analyzed during the current study are available in the GEO repository (GSE253260; 10.1016/j.ebiom.2024.105373). The RNA-sequencing datasets generated are available from the corresponding author on reasonable request.
