# Supplementary material for: Targeting FGFR4 abrogates HNF1A-driven metastasis in pancreatic ductal adenocarcinoma
Source: Mol Cancer. 2025 Jul 29;24:208. doi: 10.1186/s12943-025-02408-5 (PMC12306140; doi:10.1186/s12943-025-02408-5)

Supp Figure 1

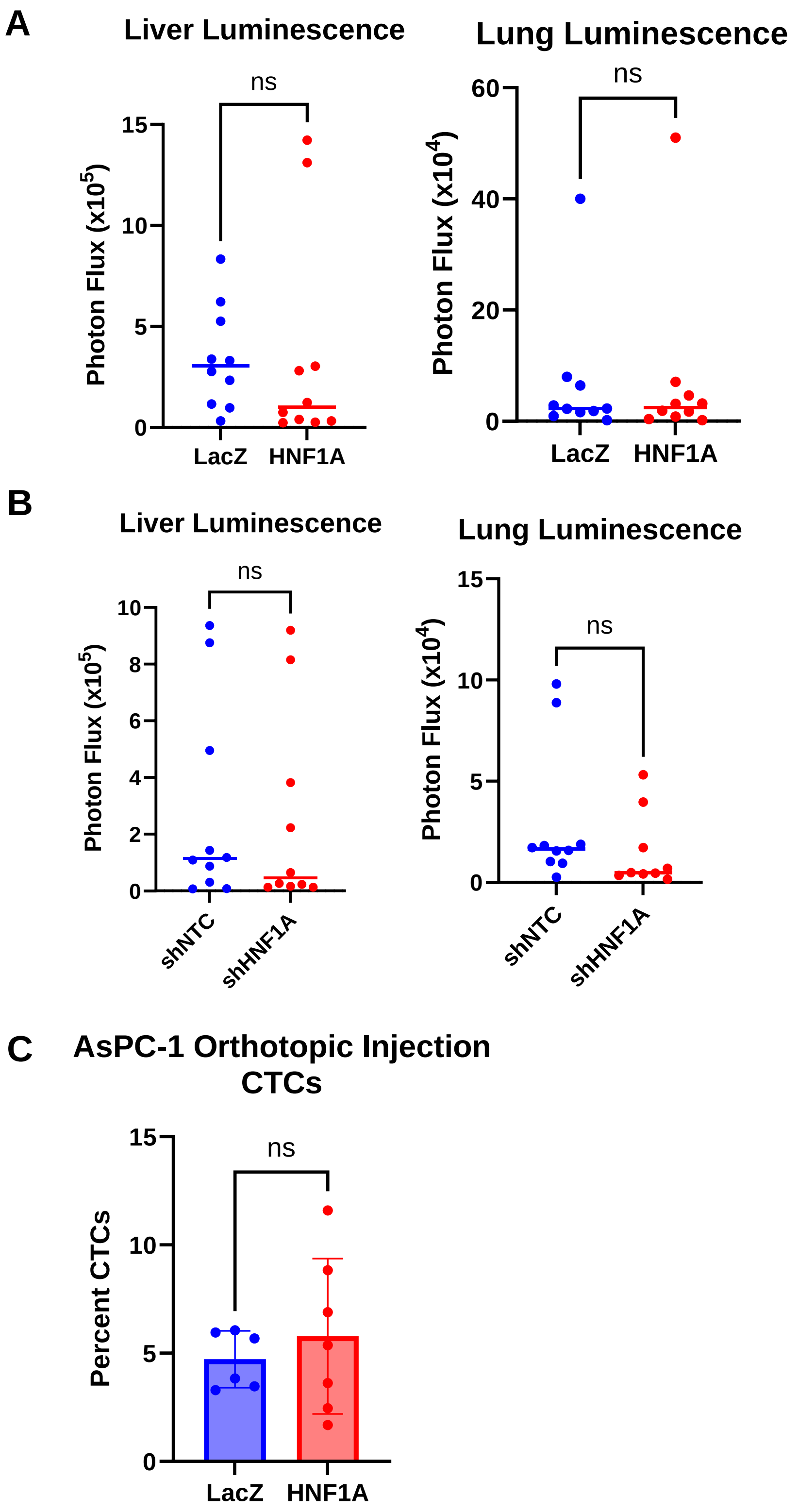

Supp Figure 2

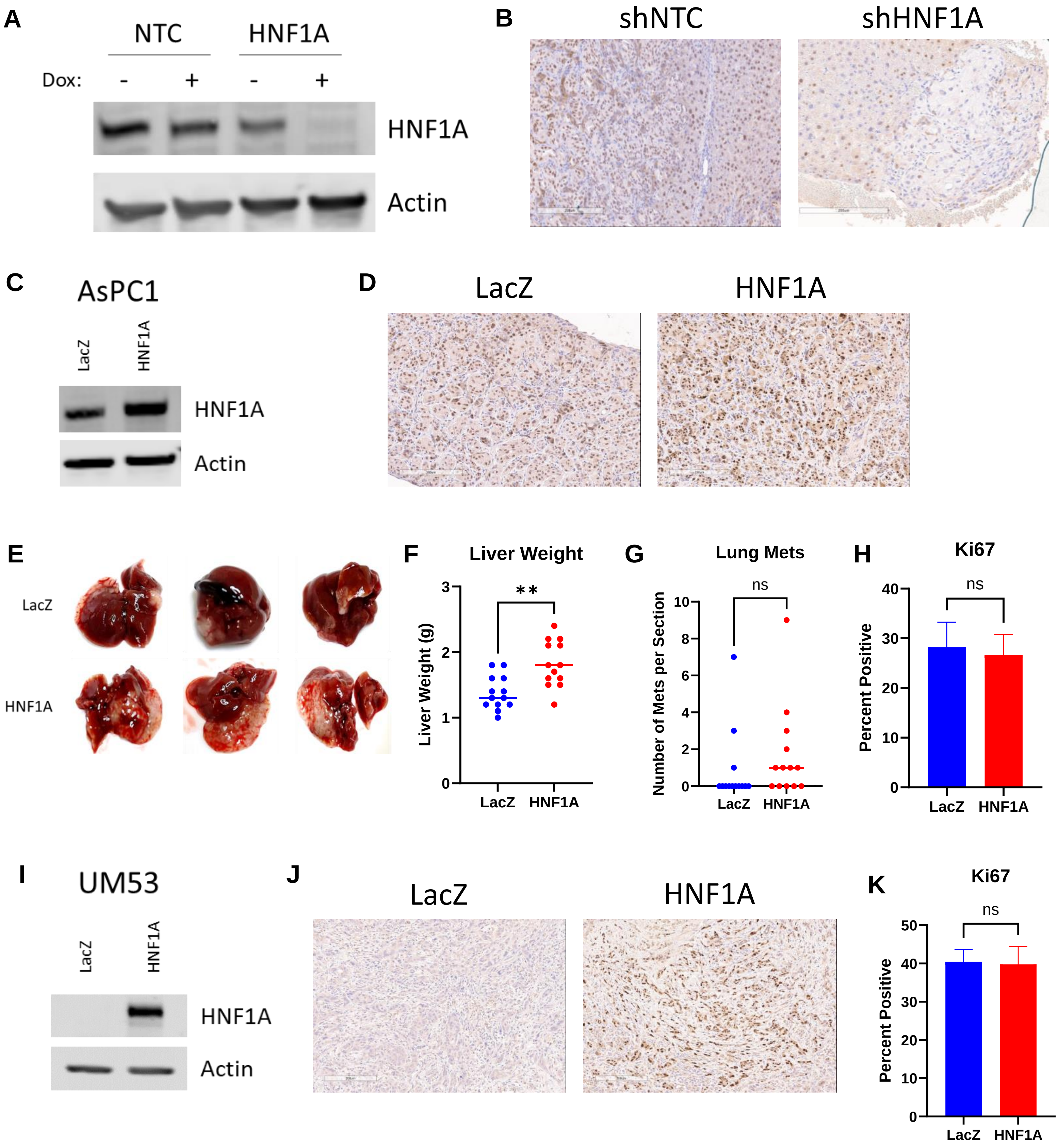

Supp Figure 3

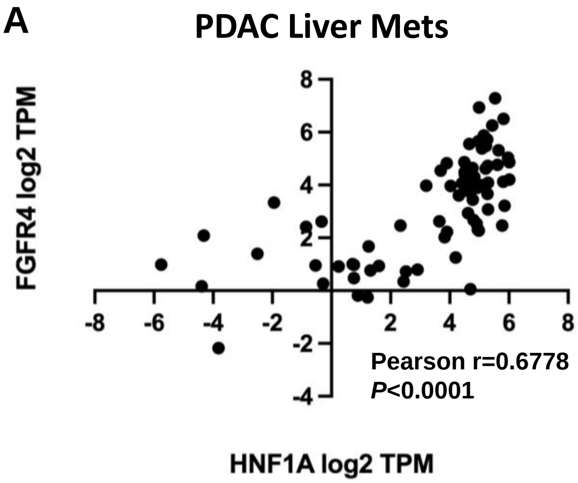

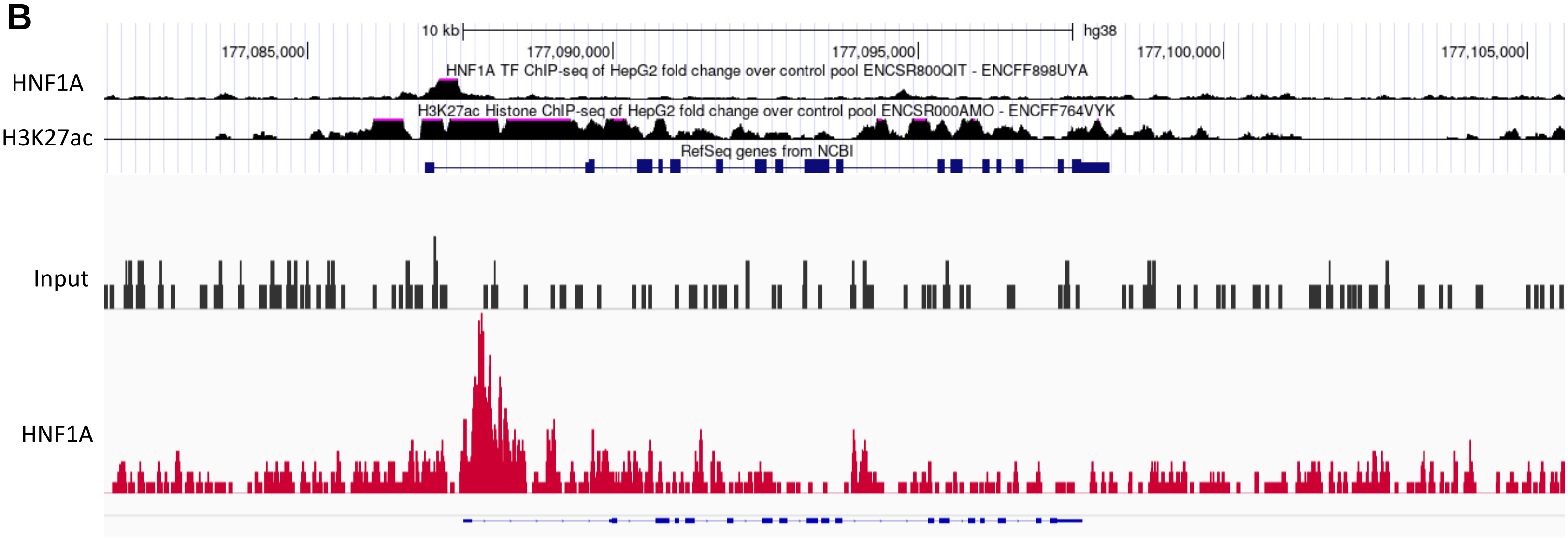

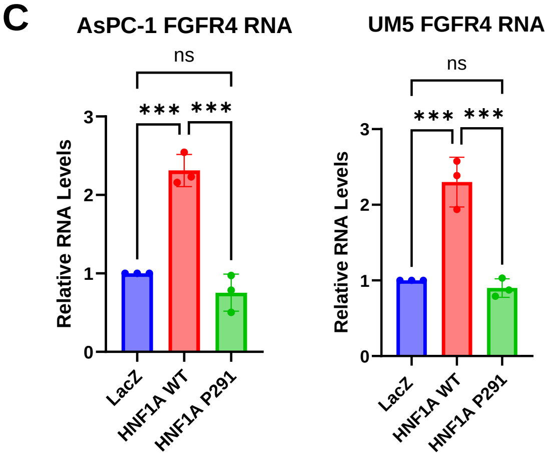

Supp Figure 4

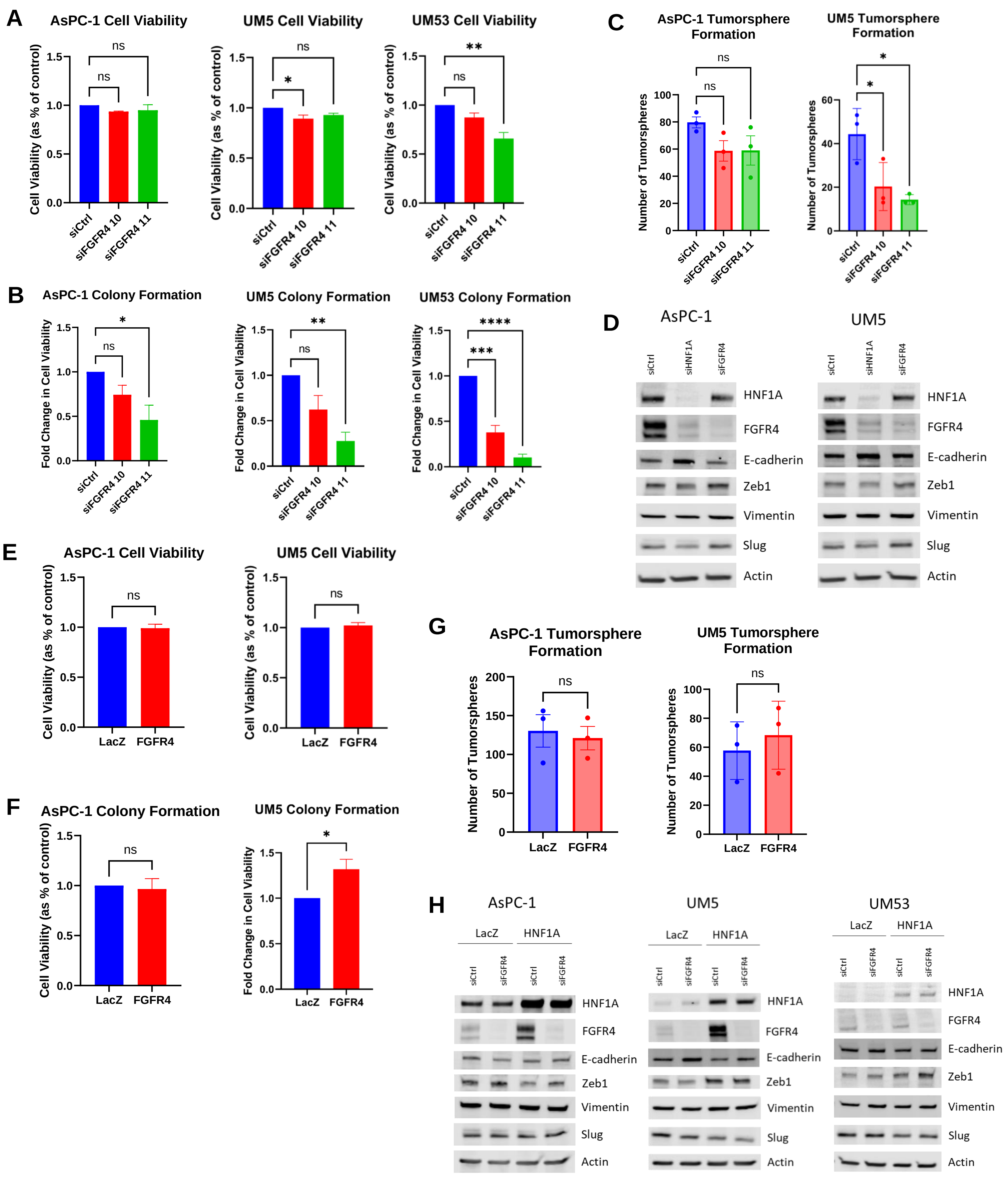

Supp Figure 5

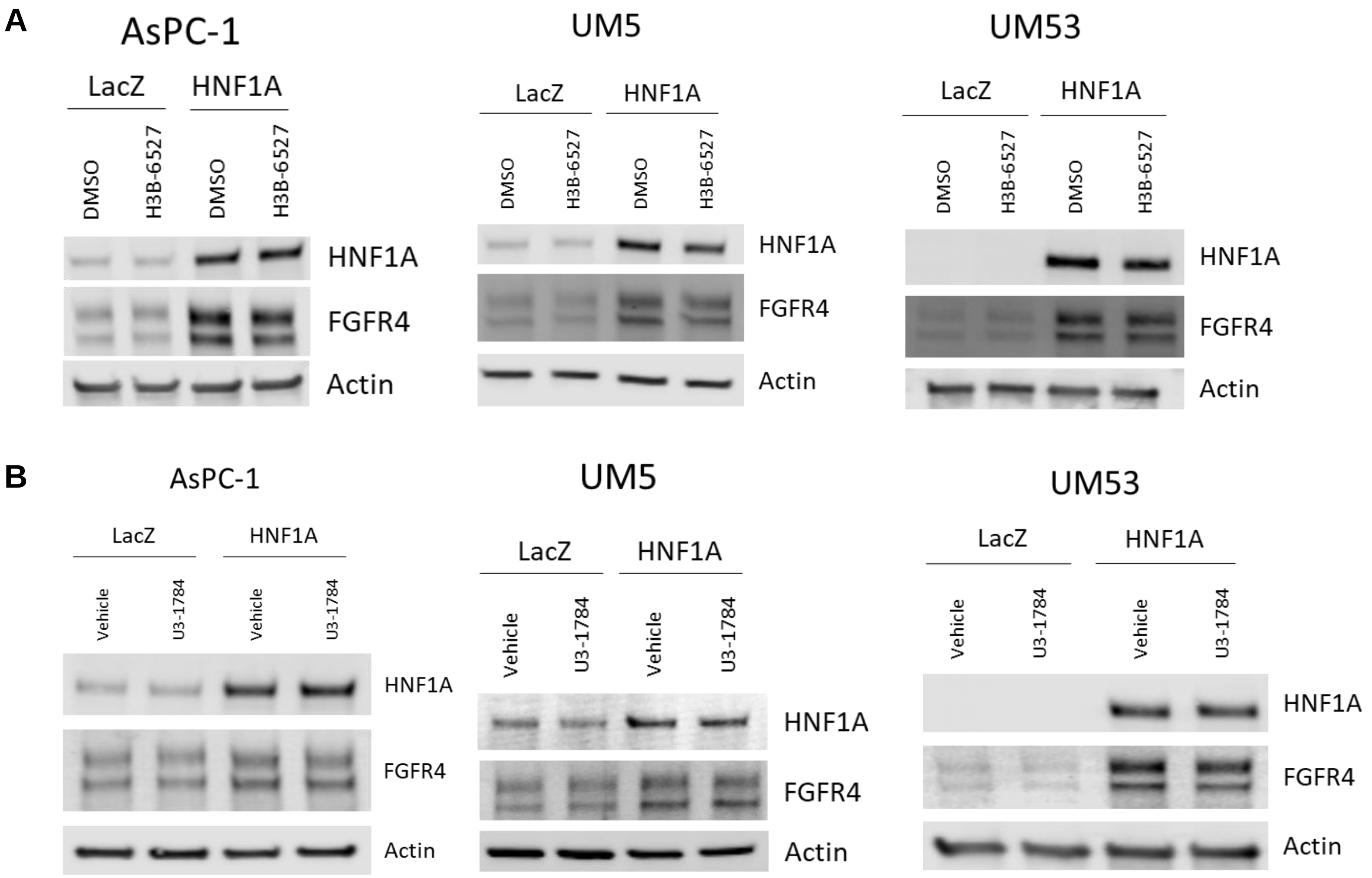

Supp Figure 6

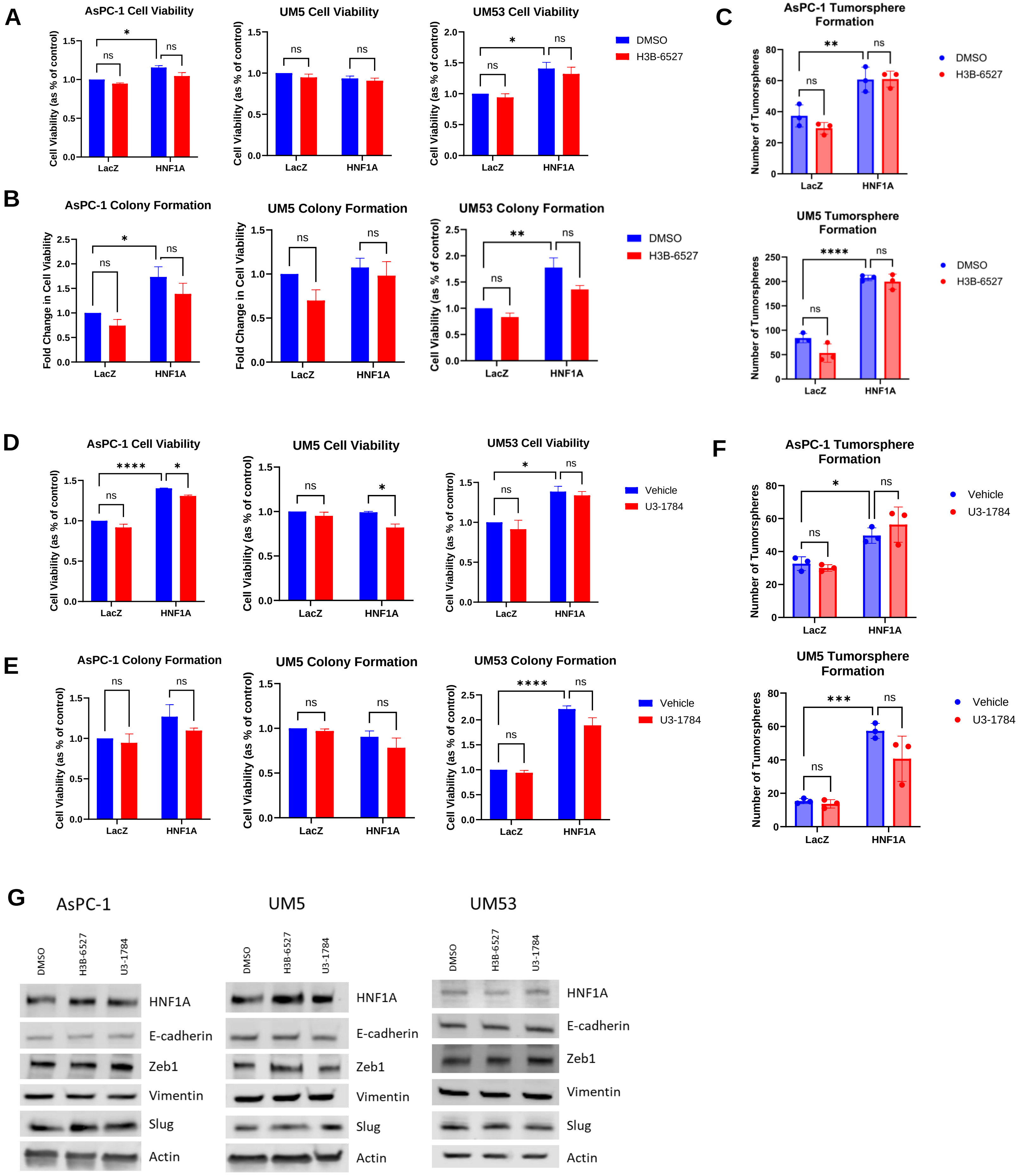

Supp Figure 7

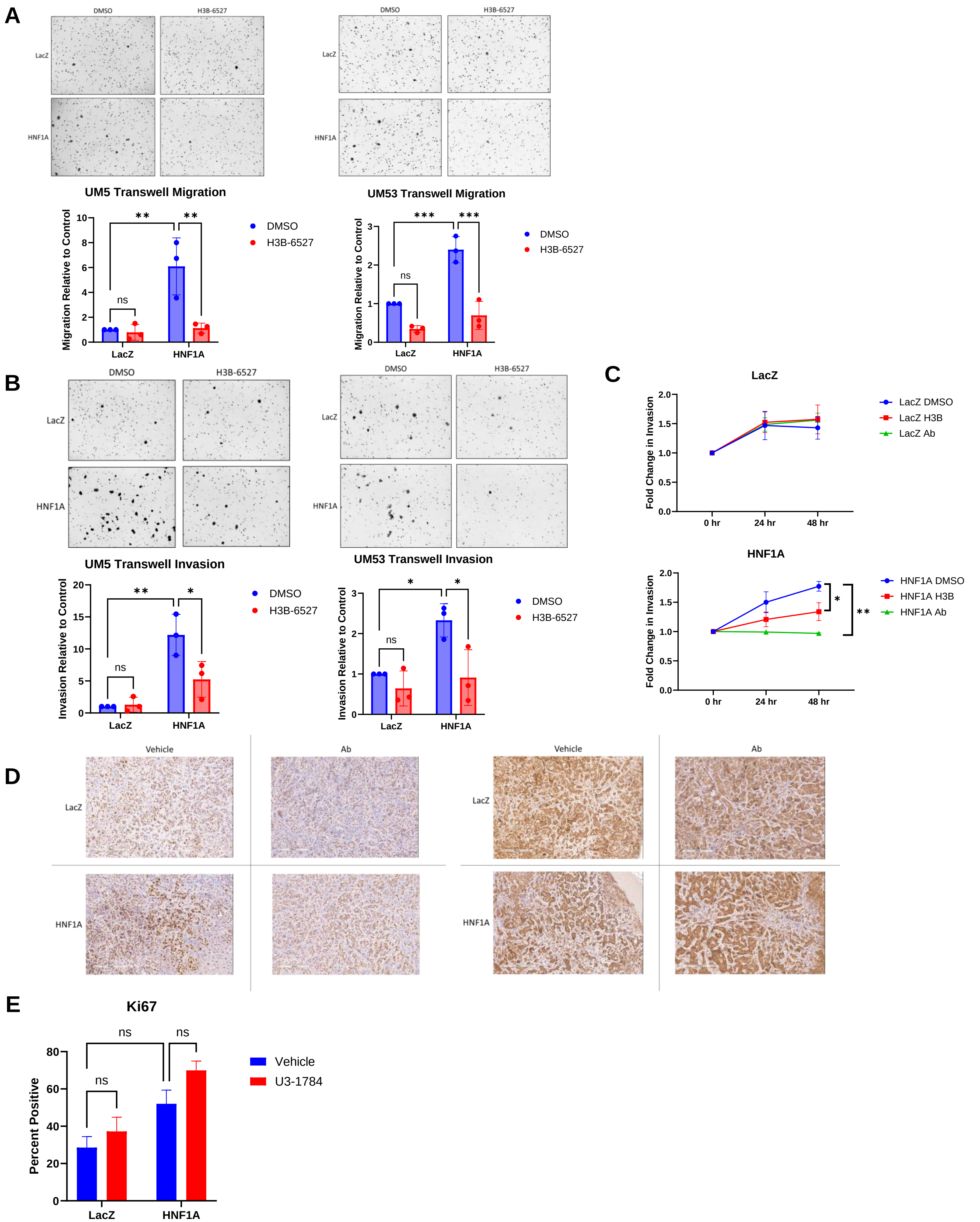

Supplement: Supplementary file 1 — Supplementary Material 1: Supp Fig. 1. A) Quantification of photon flux from bioluminescent images from all harvested livers and lungs inoculated with LacZ or HNF1A overexpressing cells at endpoint (n = 10 mice per group). B) Quantification of photon flux from bioluminescent images from all harvested livers and lungs with control or HNF1A knockdown at endpoint (n = 10 mice per group). C) Percent of blood cells positive for GFP (tumor cells) collected from the cardiac blood. All bar graphs represent the mean and statistical difference was determined by unpaired t-test. Supp Fig. 2. A) Western blotting for HNF1A with and without doxycycline administration before cell inoculation. B) Immunohistochemistry for HNF1A in the liver metastases of mice from each respective arm. C) Western blotting for HNF1A in AsPC-1 cells before inoculation. D) Immunohistochemistry for HNF1A in the liver metastases of mice from each respective arm. E) Representative photographs of harvested livers from both LacZ and HNF1A groups showing increased tumor burden (white tissue) in the HNF1A livers. F) Measured liver weights of all livers after harvest at endpoint (n = 13 mice per group). G) Quantification of the number of lung metastases per H&E stained tissue section. H) Quantification of cells positive for Ki67 immunohistochemistry staining in the liver metastases from 3 representative mice from each group. I) Western blotting for HNF1A in UM53 cells before inoculation. J) Immunohistochemistry for HNF1A in the liver metastases of mice from each respective arm. K) Quantification of cells positive for Ki67 immunohistochemistry staining in the liver metastases from 3 representative mice from each group. All bar graphs represent the mean and statistical difference was determined by unpaired t-test. Supp Fig. 3. A) Correlation of HNF1A and FGFR4 RNA expression in patient liver metastatic tumors from the European Genome Phenome Archive dataset. B) ChIP-sequencing of HNF1A and H3K27ac reads from HepG [file 12943_2025_2408_MOESM1_ESM.pdf]
